# The Drosophila gene encoding JIG protein (CG14850) is critical for CrebA nuclear trafficking during development

**DOI:** 10.1093/nar/gkad343

**Published:** 2023-05-05

**Authors:** Sayem H Bhuiyan, Guillaume Bordet, Gbolahan Bamgbose, Alexei V Tulin

**Affiliations:** University of North Dakota, Grand Forks, ND 58202, USA; University of North Dakota, Grand Forks, ND 58202, USA; University of North Dakota, Grand Forks, ND 58202, USA; University of North Dakota, Grand Forks, ND 58202, USA

## Abstract

Coordination of mitochondrial and nuclear processes is key to the cellular health; however, very little is known about the molecular mechanisms regulating nuclear-mitochondrial crosstalk. Here, we report a novel molecular mechanism controlling the shuttling of CREB (cAMP response element-binding protein) protein complex between mitochondria and nucleoplasm. We show that a previously unknown protein, herein termed as Jig, functions as a tissue-specific and developmental timing-specific coregulator in the CREB pathway. Our results demonstrate that Jig shuttles between mitochondria and nucleoplasm, interacts with CrebA protein and controls its delivery to the nucleus, thus triggering CREB-dependent transcription in nuclear chromatin and mitochondria. Ablating the expression of Jig prevents CrebA from localizing to the nucleoplasm, affecting mitochondrial functioning and morphology and leads *to Drosophila* developmental arrest at the early third instar larval stage. Together, these results implicate Jig as an essential mediator of nuclear and mitochondrial processes. We also found that Jig belongs to a family of nine similar proteins, each of which has its own tissue- and time-specific expression profile. Thus, our results are the first to describe the molecular mechanism regulating nuclear and mitochondrial processes in a tissue- and time-specific manner.

## Graphical Abstract

**Graphical Abstract:**
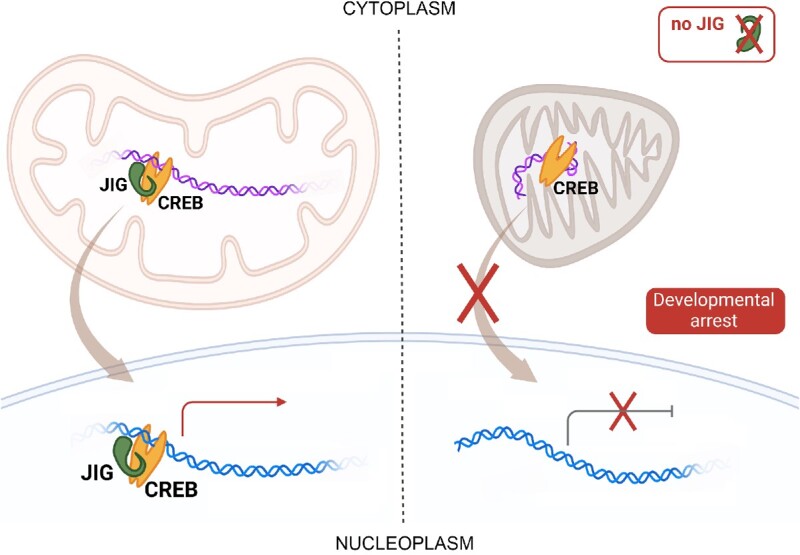
Jig (CG14850) plays a critical role during development by controlling CREB nuclear trafficking and CREB-dependent transcriptional regulation in both nucleus and in mitochondria.

## INTRODUCTION

Among roughly 1500 proteins found in animal mitochondria, only 13 polypeptides are encoded in the mitochondrial genome of animal cells, including human and fruit fly (1). The rest are encoded by the nuclear genome, translated in the cytosol and delivered to the mitochondria. As a result, many functional protein complexes localized to mitochondria are composed of protein subunits that are encoded by two different genomes. ATP synthase is a typical example of such oligomeric proteins: out of 27 of its subunits, two are encoded in mitochondria, and the rest—by the nuclear genome (2). Thus, the production of proteins encoded by the nucleus and the mitochondria should be coordinated. Without proper coordination between nuclear and mitochondrial gene expression and the molecular mechanism that establishes mitochondrial-nuclear crosstalk, no ATP synthase and respiratory complexes can be properly generated, leading to a buildup of non-working subunits that would cripple cellular energetics. The breakdown of such crosstalk between two organelles leads to mitochondrial dysfunction, which results in severe health issues, such as neurodegenerative diseases and cancer (3,4).

Mitochondrial and nuclear communication is thought to occur through transcription factors (TFs) or coactivators that regulate both mitochondrial and nuclear gene expression (5). These transcription factors and coactivators can be activated by external stimuli, such as changes in surrounding temperature, exercise, and food intake, as well as internal changes of certain hormone levels (6–9). The nucleus regulates mitochondrial biogenesis through these TFs (10). Nuclear Respiratory Factor 1 (NRF1), for example, activates transcription of many nuclear-encoded mitochondrial genes involved in mitochondrial biogenesis, including mitochondrial transcription factor A (TFAM) (11). Previous studies on nuclear-encoded mitochondrial genes also led to the discovery of TF-like Nuclear Respiratory Factor 2 (NRF2) and cAMP response-element binding protein (CREB) that regulate the expression of nuclear-encoded mitochondrial proteins (11). Translocation of such proteins from the cytosol to the nucleus and back, followed by the association of such proteins with mitochondria, have a coordinated effect on nuclear transcription and mitochondrial biogenesis, including mitochondrial fission, fusion, and protein translocation. While this type of communication, ‘anterograde signaling’ from the nucleus to the mitochondria, has been described as a major route of communication called between the organelles (12), another mechanism of communication called ‘retrograde signaling’, when mitochondria release Ca^2+^ into the cytoplasm, also takes place, thereby triggering signaling pathways that activate nuclear transcription factors, such as CREB, NFkB and ATF2, that activate genes responsible for regulating mitochondria and/or reducing mitochondrial stress (5,13).

Apart from the mechanisms described above, there is a rare scenario with only a handful of proteins that have been confirmed to reside in both, the nucleus and the mitochondrial matrix, and directly interacting with both genomes (12). All such dual-localized TFs and coactivators do not account for the regulation of all the nuclear genome-encoded mitochondrial genes (5), leaving a big gap of knowledge that prevents the full understanding of the nuclear-mitochondrial communication pathway that coordinates the expression of both genomes. Identification and study of more of these rare dual-localized proteins would give us a better understanding of the molecular mechanisms underlying mito-nuclear communication, as well as the impact on energetics.

Here we showed, for the first time, that a novel protein, CG14850, localizes to nuclear chromatin and in mitochondria in *Drosophila melanogaster*. We named the CG14850 gene product ‘Jig’ after the small fishing bait device, which is similar to the ribbon structure of Jig and is designed to hook something and pull (here and thereafter CG14850 will be referred as ‘Jig’ gene). Using confocal microscopy, we showed that Jig binds to nuclear chromatin. Using a genome-wide ChIP-seq approach, we have confirmed the association of Jig with nuclear chromatin at specific loci and found that Jig binds mitochondrial and nuclear chromatin. Genes, bound by Jig in both nuclear and mitochondrial genomes, were also identified. Co-immunoprecipitation revealed that Jig interacts with one of the dual-localized transcription factors, CREB, that plays an important role in the nuclear-mitochondrial communication pathway. Chromosome squash, followed by confocal microscopy experiments, confirmed functional and physical association of Jig with *Drosophila* CREB (CrebA) in the nuclear chromatin.

## MATERIALS AND METHODS

### Drosophila strains and genetics

Genetic markers are described in FlyBase (14), and stocks were obtained from the Bloomington Stock Center, except as indicated. pP{w1, UAST::Tim17B-DsRed}(10), called Tim17B-DsRed, was described in (15). The following GAL4 driver strains were used: 69B-GAL4 (16) and Arm::GAL4 (Bloomington stock #1560). Balancer chromosome carrying Kr::GFP, i.e. FM7i, P{w1, Kr-GFP}, was used to identify heterozygous and homozygous transgenic animals (17). siRNA transgenic *Drosophila* stocks #48673 (siRNA1) and #104550 (siRNA2) were obtained from VDRC (18).

### Construction of transgenic drosophila

**Figure 1.**
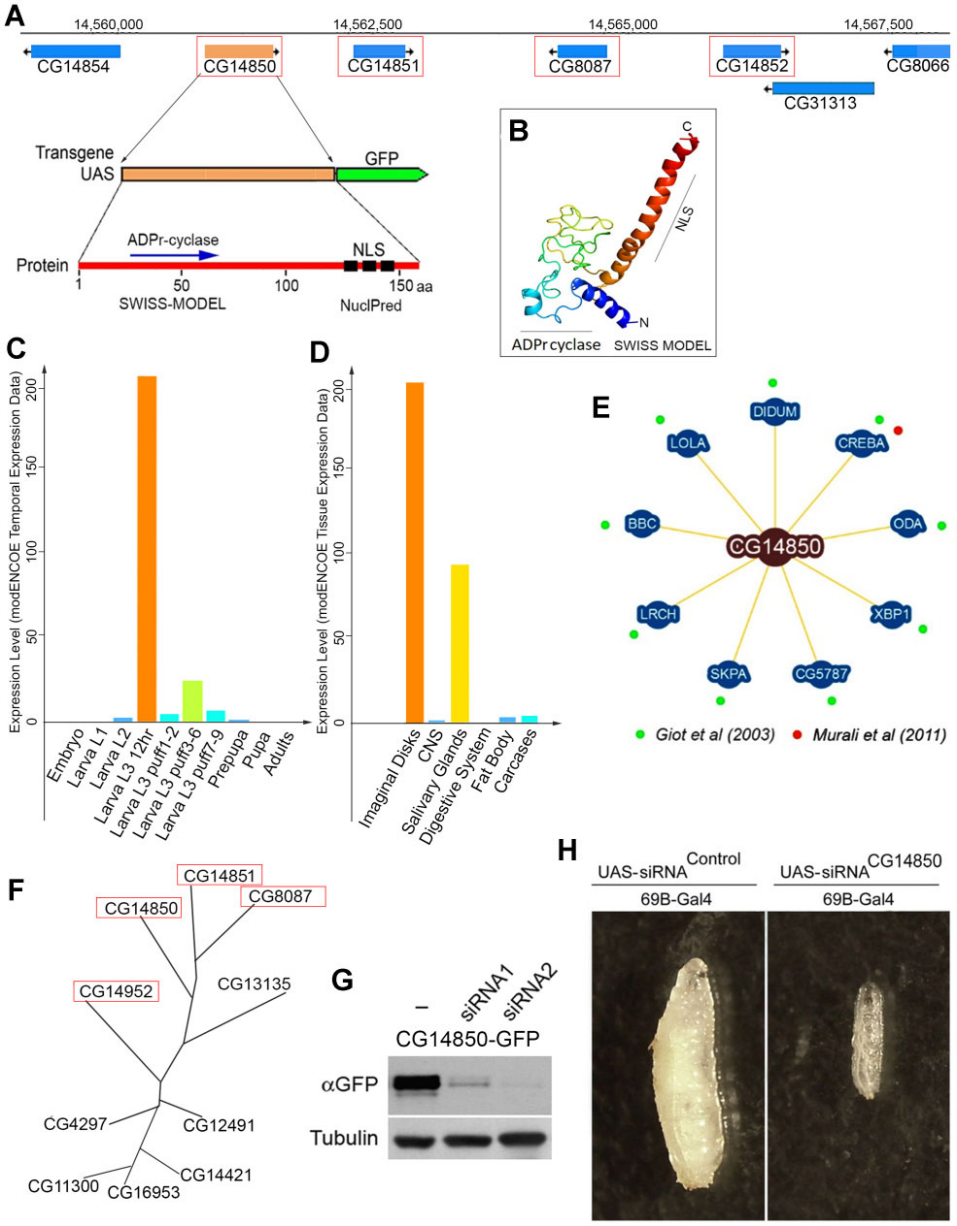
*D. melanogaster* gene CG14850 encodes Jig that plays vital roles in development. (**A**) Structure of the genomic locus encoding Jig (above); structure of GFP-tagged Jig transgenic reporter (middle); predicted structure of Jig protein (below) (see also Supplemental Figure S1). Red frames indicate paralogues of Jig. (**B**) Three-dimensional structure of Jig protein predicted by the SWISS MODEL (35) and Phyre square software (36). (**C**) Jig protein expressed from embryo to adult stages. Data obtained from the mod ENCODE Temporal Expression Data Project (37). (**D**) Jig protein expressed almost exclusively in larval imaginal discs and larval salivary glands. Data obtained from the mod ENCODE Tissue Expression Data Project (37). (**E**) Two-hybrid approach identified nine proteins interacting with Jig protein in *Drosophila* (38,39). (**F**) Evolutionary tree of Jig paralogs in the *D. melanogaster* genome (see also Supplemental Figure S2). Red frames indicate paralogues of Jig located in the same genomic locus. (**G**) Knockdown-transgenes eliminate Jig-GFP protein expression in *Drosophila*. Two different siRNA constructs against Jig were expressed using 69B-GAL4 driver in Jig-GFP-expressing *Drosophila*. Total protein extracts from third instar larvae were subjected to Western blot analysis using anti-GFP antibody. LexA siRNA-expressing animals of the same genetic background were used as a control. Tubulin antibody was used as a loading control. (**H**) Jig is required for *Drosophila* development. siRNA against Jig was expressed using 69B-GAL4 driver in wild-type *Drosophila*. All Jig siRNA-expressing animals were arrested in early third instar larval stage. LexA siRNA-expressing animals of the same genetic background were used as a control.

To construct UAS::Jig-GFP, we generated the full-length genomic fragment of Jig locus (Figure 1A) using PCR. We used wild-type *Drosophila* genomic DNA as a template for PCR. The resulting PCR products were cloned through The *Drosophila* Gateway™ Vector Cloning System (Carnegie Institution of Washington) into the corresponding vector for *Drosophila* transformation. Generating transgenic *Drosophila* was performed as described (19,20).

### Western blot

The following antibodies were used for immunoblotting assays: anti-B-actin (Mouse monoclonal, 1:5000, Sigma, #A5441), anti-Tubulin (Mouse monoclonal, 1:20 000, Sigma, B512), anti-CrebA antibody (Rabbit polyclonal, 1:2000, DSHB, AB_10805295) and anti-GFP (Mouse monoclonal, BD, #632380, 1:4000). Western blotting was done using the detection kit from Amersham/GE Healthcare (#RPN2106), according to the manufacturer's instructions.

### Drosophila salivary gland polytene chromosome immunostaining

Preparation and immunostaining of polytene chromosome squashes were performed exactly as described (21). The primary antibody used was anti-GFP (Rabbit, Torrey Pines Biolabs, #TP401, 1:400), and the secondary antibody used was goat anti-rabbit Alexa-488 (Molecular Probes (1:1500)). Anti-CrebA antibody was also used (Rabbit polyclonal, 1:2000, DSHB, AB_10805295). Slides were mounted in Vectashield (Vector Laboratories, Burlingame, CA) with propidium iodide at 0.05 mg/ml for DNA staining.

### Whole mount drosophila tissue immunohistochemistry

Third instar larvae of the appropriate stages were collected prior to dissection. Tissues were dissected in Grace's insect medium, fixed in 4% paraformaldehyde + 0.1% Triton X-100 in PBS for 20 min, and blocked with 0.1% Triton X-100 + 1% BSA for 2 h. These tissues were then incubated with primary antibody overnight at 4°C, washed three times with PBS + 0.1% Triton X-100, and then incubated with fluorescence-labeled secondary antibody Alexa Fluor 488, 568 or 633 goat anti-mouse or anti-Rabbit (1:1500; Invitrogen) for 2hrs at room temperature. After washing three times with PBS + 0.1% Triton X-100, DNA was stained with TOTO™-3 Iodide (642/660) antibody (1:3000, T3604, Fisher). Slides were mounted in Vectashield (Vector Laboratories, Burlingame, CA).

### Colocalization analysis

Colocalization analysis was performed on ImageJ (22) using the Colocalization_finder plugin (available at this link: http://questpharma.u-strasbg.fr/html/colocalization-finder.html). A 512 × 512 scatterplot was generated based on the CrebA or Jig fluorescence intensity, along chromosome piece. The Pearson correlation coefficient was calculated based on this scatterplot.

### MitoTracker red staining

Salivary glands from early third instar larvae were obtained through dissection at room temperature. in Phosphate Buffer Saline (PBS). After a short PBST (0.1%) wash, samples were incubated for 5 min in 100 nM MitoTracker RedCMXRos. After three short washes with PBST (0.1%), the samples were fixed in 4% paraformaldehyde in PBST at room temperature. for 1 min (23).

### Sample preparation for ChIP-seq

Flies were bred in a tube for an 8-h period, and the eggs laid were allowed to grow at room temperature. The third instar larvae at 12 h stage were collected using 15% sucrose solution. About 0.20 g of larvae was collected. Larvae were washed with 1ml of 1× PBS by spinning them down at 10 000g. Larvae were homogenized with pestle in 800 ul 1× PBS, 10ul Protease inhibitor cocktail, 1 ul Tween-20 and 250 ul of PMSF. Formaldehyde was added to 1.8%. Sample was crosslinked on a rotator at r.t.p. for 15 min. Crosslinking was quenched by adding 500 mM of Glycine. The quenched sample was incubated on ice for 5 min. Larval cells were then centrifuged at 1000g for 3 min, the supernatant discarded, and the pellet suspended in 1 ml of Sonication buffer (0.5% SDS, 20 mM Tris, pH 8.0, 2 mM 0.5 M EDTA, 0.5 mM EGTA, 0.5 mM PMSF and 100× Protease inhibitor cocktail). Samples were then sonicated using the Bioruptor sonication machine for 20 cycles. Sonicated samples were centrifuged at 10 000g for 10 min, and supernatant was collected.

After overnight decrosslinking, 750 ul phenol/ chloroform/isoamyl were added to the samples and were vortexed and centrifuged at 10 000g. The top layer was collected, and DNA was precipitated with 1ml of 100% ethanol. Samples were centrifuged at maximum speed for 20 min. Washing was done using 70% ethanol, and centrifuging was performed at maximum speed for 5 min. The pellet was suspended in 22 ul of nuclease-free water.

For each IP, 10% of the sample was used for input. Each IP was diluted to a volume of 1 ml using IP buffer (0.5% Triton X-100, 2mM of EDTA, 20 nM of Tris–HCl pH 8, 150 nM NaCl and 10% glycerol). 100 ul Agarose A beads (50% slurry with IP buffer) were added to each IP. IPs were then rotated at 4°C for 1 to 2 h. They were centrifuged for 1 min at 1000g. 250 ul of IP buffer and 5 ul of anti-GFP antibody were added to the supernatant. The IPs were rotated overnight at 4°C. 200 ul of protein-A agarose (50% slurry) were added, and IPs were centrifuged at 1000g in 4°C for 1min. Pelleted beads were washed with 1ml of low salt buffer (0.1% SDS, 1% Triton X-100, 2 mM EDTA, 20 mM Tris–HCl, 150 mM NaCl), rotated for 4 min at r.t.p., and centrifuged for 1 minute at 1000g. This washing procedure was repeated 3 times with high salt buffer (0.1% SDS, 1% Triton X-100, 2 mM EDTA, 20 mM Tris–HCl, 500 mM NaCl), 1 time with LiCl buffer (2 mM EDTA, 20 mM Tris–HCl pH 8.0, 0.25 M LiCl and 1% NP-40), and 2 times with 1 ml TE buffer (10 mM Tris–HCl pH8.0 and 1 mM EDTA). DNA from the IPs and Inputs were eluted using 250 ul of elution buffer (1% SDS, 100 mM NaHCO_3_).

Decrosslinking was done overnight at 65°C. 15 ul of 1 M Tris–HCl (pH 7.5), 2 ul Glycoblue and 2 ul Proteinase K were added to each sample and incubated at 65°C for 30 min. DNA was extracted using 750u l of phenol/chloroform/isoamyl. DNA was sent to Novogene for library preparation and sequencing.

### ChIP-seq data analysis

ChIP-seq analysis was done using the web-based Galaxy platform. Paired-end reads were mapped against the DM6 *Drosophila* melanogaster genomic database. Peak calls were done using the MACS2 callpeak tool with default parameters in Galaxy. Distribution of Jig binding sites relative to TSS was generated using the plotheatmap tool in Galaxy with a parameter range set to –3 and +3 kb from TSS (24).

Gene Ontology analysis was done with the String application using the Jig binding gene list (25). List of active and inactive genes during the L3 12 h stage was obtained from Flybase (14). These gene lists were compared with the list of Jig-bound genes using Excel to determine percent of Jig-bound genes that were active genes and inactive.

Human orthologs of Jig bound genes were obtained using DIOPT Ortholog Finder from Harvard Medical School (26). Human CREB target lists were obtained from the CREB transcription factor datasets collected by Harmonizome from Encode Transcription Factor Targets Database (27,28).

Raw data for *Drosophila* CrebA chip-seq were obtained from the Encode Database (28). These publicly available deposited data were generated from ChIP-seq on 3^rd^ instar *Drosophila* melanogaster larvae with anti-GFP antibody against CrebA-eGFP. CrebA binding site distribution, target profile and gene ontology data were generated using the same method as that used for Jig.

### JC-1 mitochondrial membrane potential assay

3rd instar *Drosophila* larvae at 6hrs stage were dissected in HL-3 buffer (70 mM NaCl, 5 mM KCl, 20 mM MgCl_2_, 10 mM NaHCO_3_, 115 mM sucrose and 5 nM HEPES, pH 7.2). JC-1 dye was added to HL-3 buffer at 1:800 dilution, and each dissected larva was incubated in the solution for 10 min. Samples were then washed for 5mins using HL-3 buffer twice and mounted for imaging. Samples were excited at wavelengths of 488 and 555 nm for green and red fluorescence, respectively (29).

### Electron microscopy

For ultrastructural analysis, the salivary glands were dissected, fixed in 2% formaldehyde/ 2% glutaraldehyde in 0.1 M cacodylate buffer pH 7.2, post-fixed in 1% OsO_4_, dehydrated in ethanol and propylenoxide, and embedded in EMbed-812 (EMS, Fort Washington, PA) in flat molds. After polymerization for 60 h at 65°C, 70nm sections were cut on a Leica Ultracut E microtome (Leica, Austria), placed on collodion/carbon-coated grids, and stained with 2% uranyl acetate/lead citrate. Sections were viewed on a Tecnai 12 transmission electron microscope (TEM) (FEI, Hillsboro, OR). For EM immunocytochemistry, samples were prepared according to Tokuyasu (1980) (30). In brief, the dissected salivary glands were fixed in 4% formaldehyde/0.2% glutaraldehyde in 0.1M PHEM (60 mM PIPES, 25 mM HEPES, 2 mM MgCl_2_, 10 mM EGTA, pH 6.9), cryo-protected in 2.3 M sucrose, mounted on aluminum pins, and frozen in liquid nitrogen. Thin frozen sections were then cut on a Leica EM UC6/FC6 cryo-microtome (Leica, Austria), collected on a drop of sucrose/methylcellulose mixture and placed on a formvar-carbon grid. The sections were labeled with primary antibody, and the label was subsequently visualized by colloidal gold conjugated to Protein A. Sections were stained/embedded in 2% methylcellulose/0.2% uranyl acetate and observed under a Tecnai 12 TEM.

### Co-immunoprecipitation assay

Lysates for immunoprecipitation were prepared as follows: 15 third-instar larvae were collected for each sample. They were put into Eppendorf tubes and rinsed 3 times with 1 ml of dist. water. 500 ul of ice-cold lysis buffer (10 mM Tris–HCl pH7.5, 150 mM NaCl, 1 mM EDTA, 0.2% NP40, 1% Triton X100, 0.1% SDS, 1% sodium deoxycholate, Complete™ protease inhibitors (Roche) and 0.1 mM Pefabloc SC (Fluka) were added to each tube, and larvae were homogenized by hand pestle homogenizer on ice. After 30min incubation on ice, samples were centrifuged at 14 500 RPM for 20 min (4°C). Supernatants were transferred to new Eppendorf tubes on ice. For one immunoprecipitation reaction, 500 mkl of total lysates were incubated with 25 ml Protein-G Sepharose 4B (Sigma #P3296-5ML) on a rotating platform for 1 h 30min at 4°C. Beads were removed by spinning 1 min at 2000g. An appropriate amount of antibody was added to the lysates, and the mixture was incubated 4hrs on a rotating platform at 4°C. The following antibodies were used for immunoprecipitation: anti-GFP (JL8). Then, 30 ul of Protein-G Sepharose 4B were added to the lysates and incubated overnight at 4°C with rotation. Beads were washed 4 times for 5 min in 1.0 ml of the lysis buffer. Bound proteins were eluted by 60 ml of 1× Laemli with heating at 95°C for 5min.

### Third instar larval staging

The start of L3 12 h is defined as the period right after molting into third instar from second instar. This period is ended by a mid-level ecdysone spike during the middle stage of *Drosophila* third instar larval development. This marks the start of puffing stage 1–2 at which time the third instar larvae start wandering out of the food. This time, the larvae have a dark blue gut. This is the mid-stage of third instar larval development. The latest portion of third instar larval development is puff stage 7–9 where different genes in *Drosophila* salivary glands become puffed. Larvae have clear guts at this point. This period is ended by a major ecdysone peak causing the larvae to form pre-pupae (31,32).

### RNA extraction followed by reverse transcriptase qPCR

This assay was performed in triplicate. Twenty third instar larvae were collected for three groups (siRNA Control, siRNA1 JIG and siRNA2 JIG). Total RNA was extracted from cells using the QIAshredder column and RNeasy kit (Qiagen). Contaminating genomic DNA was removed by the g-column provided in the kit. cDNA was obtained by reverse transcription using M-NLV reverse transcriptase (Invitrogen). Real-time PCR assays were run using SYBR Green master mix (Bio-Rad) and an Applied Biosystems StepOnePlusTM instrument. The amount of DNA was normalized using the difference in threshold cycle (CT) values (ΔCT) between rpL32 and Jig targets.

The quantitative real-time PCR (qPCR) primer sequences for Drosophila melanogaster ribosomal protein L32 gene (rpL32) were 5’-GCTAAGCTGTCGCAACAAAT-3’ (forward) and 5’-GAACTTCTTGAATCCGGTGGG-3’ (reverse).

Sequences for Jig targets were

- mt-CoI (Forward) GACTTCTACCTCCTGCTCTTTC
- mt-CoI (Reverse) CAGCGGATAGAGGTGGATAAAC
- mt-CoIII (Forward) TCTACACACTCAAATCACCCTTT
- mt-CoIII (Reverse) TATAGCTCCGATAGCTCCTGTT
- ccz1 (Forward) GAAGGCGAGGAACACAAGAAG
- ccz1 (Reverse) AGTCCCACATCTTTGATTTTCGT
- Cyt-c-d (Forward) TCTGGTGATGCAGAGAACGG
- Cyt-c-d (Reverse) CACTTCGTAGGTGTGGCACT
- Surf1 (Forward) AAAGATGACACAACAGCGACC
- Surf1 (Reverse) GGAACCATCCCAAAGGAGCTA
- ROS levels in third instar larvae tissues

To evaluated ROS levels we used MitoSOX™ Mitochondrial Superoxide Indicators, for live-cell imaging (Catalog number: M36006).

### NAD and NADH levels in third instar larvae tissues

We used the ScienCell Research Laboratories NAD/NADH assay (Cat. # 8368).

### Motif analysis

Find Individual Motif Occurrences (FIMO) (33) was used to determine the occurrence of CrebA consensus motif (34) at Jig binding sites within promoters (±1000 bp TSS) using a *P*-value threshold <0.001.

### Confocal imaging and quantification

Prepared slides were mounted on the Leica TCS SP8 confocal microscope stage and viewed under 63× optical lens. Using lasers, samples were excited at 488, 552 and 638 nm to detect fluorescence from GFP, CrebA and TOTO3 DNA stain respectively. The whole cell area was chosen, and the fluorescence was recorded using ImageJ. The nuclear area from samples were chosen and fluorescence was recorded. Fluorescence from the nucleus was subtracted from fluorescence recorded from the whole area to get the fluorescence coming from the cytoplasm.

To calculate the distribution of JIG and CREB proteins between the nucleus and cytoplasm, we stained dissected salivary glands with DNA marker TOTO3 and using the appropriate antibodies. Confocal images of the whole organs were taken on Leica DMI8 confocal system and then analyzed using QuPath 0.4.0 software (54). We utilized the deep-learning neural network StarDist trained to detect fluorescently labeled nuclei (55,56). Nuclei were detected in TOTO3 channel (633 laser), and fluorescence intensity was calculated for proteins (JIG and CREB) in 488 laser channel. We defined the cells as area 10mkm around each detected nucleus. The ratio in protein signal between the cytoplasm and nucleus was be calculated for the whole organ.

## RESULTS

### Jig encodes a 19kDA protein that controls *drosophila* development

The *Drosophila* Jig protein is encoded by a small intron less gene located in the third chromosome (Figure 1A, Supplemental Figure S2). Jig protein contains 158aa with a nuclear localization signal located at the C-terminus (Figure 1A, B, Supplemental Figure S1). The Swiss Model (35) and Phyre square software (36) predicted a 3D model of Jig protein with local protein folding similar to that of the ADPr-cyclase protein (Figure 1B). The modENCODE Tissue and Temporal Expression Project (37) data analysis demonstrates that Jig expression is limited to a very short developmental stage, 3rd instar larvae (Figure 1C), and that it is almost exclusively limited to precursors of adult tissues, imaginal disks, and larval salivary glands (Figure 1D). Previous genome-wide studies (38,39) reported that CG14850 protein product (Jig) potentially interact with nine *Drosophila* proteins, including transcriptional factor cyclic-amp response element binding protein A (CrebA), which is involved in nuclear genome regulation (40) (Figure 1E). Other Jig putative interactors include two other transcriptional factors called Longitudinals Lacking (LOLA) and X Box Binding Protein 1 (XBP1); two cytoskeleton-associated proteins called Dilute Class Unconventional Myosin (DIDUM) and CG5787; component of protein-degradation machinery, SKP1-related A (SKPA); cyclic nucleotide-binder, Leucine-Rich-Repeats and calponin homology domain protein (LRCH), as well as a component of phospholipid biosynthesis, bb in a Boxcar (BBC) (31).

Owing to its very small size, extensive search does not reveal any obvious homologs to Jig, other than in *Drosophila*e genomes. In the *Drosophila melanogaster* genome, Jig has nine paralogs (Figure 1F, Supplementary Figure S2), four of which are located on the same chromosomal locus (Figure 1A). Four paralogues, Jig, CG14851, CG8087 and CG13135, share almost all Jig protein features (Supplemental Figure S2A), including five conserved cysteines, suggesting that these proteins are either involved in protein-protein interactions via disulfide bounds or have Zn-finger-like structural domains.

To study functions and localization of Jig *in vivo*, we created a transgenic reporter construct by fusing the Jig with a C-terminal green fluorescent protein (GFP) tag under control of inducible UASt promoter (Figure 1A), and we generated transgenic flies expressing the fusion protein (Figure 1G). The Jig-GFP fusion does not change the phenotype of these flies. They are reproducing, healthy and viable. We also used knockdown transgenic constructs to produce two different nonoverlapping siRNAs. In our control experiment, the expression of these siRNAs effectively eliminated Jig-GFP protein expression (Figure 1G). The ubiquitous expression of these RNAi transgenes in wild-type *Drosophila* arrests the fly's development at early 3rd instar larval stage (Figure 1H), but does not cause immediate lethality. These observations indicate that Jig has a vital function during *Drosophila* development.

### Jig protein localizes to nuclear chromatin and mitochondria

**Figure 2.**
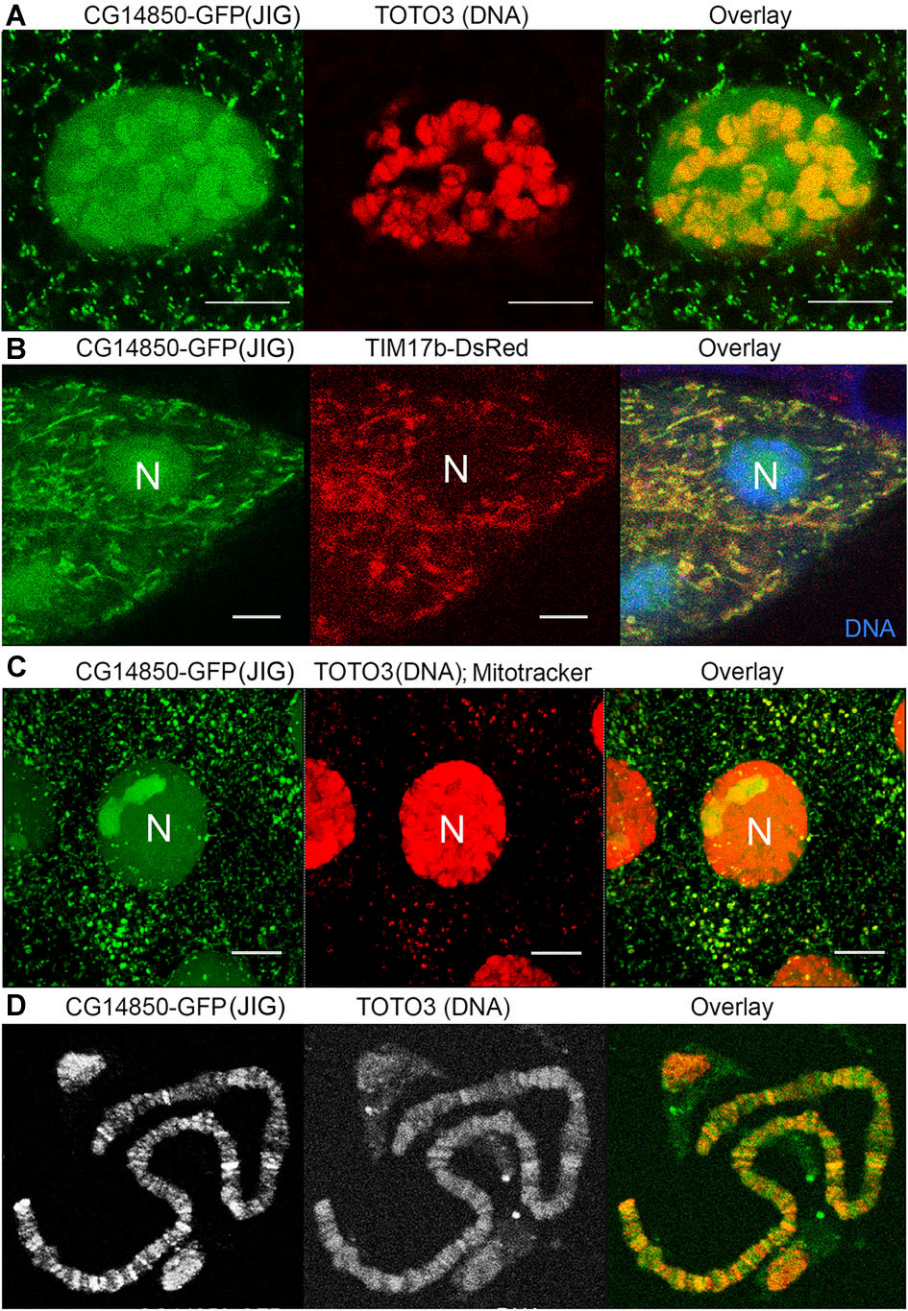
Jig protein localizes to mitochondria and nuclear chromatin in *Drosophila* third instar larvae cells. (**A**) Jig protein has a dual nuclear-cytoplasmic localization. Jig-GFP (Green) transgene was expressed using 69B-GAL4 driver in wild-type 12 h third instar larvae. A single salivary gland cell is shown. DNA was detected using TOTO3 (red) dye staining. (**B**) Jig protein is localized to mitochondria. Third instar larval salivary glands expressing Jig-GFP (green) and mitochondrial protein TIM17B-DsRed (15) (red) were stained with TOTO3 (Blue) to stain nuclear chromatin. (**C**) Jig protein is localized to mitochondria. Third instar larval salivary glands expressing Jig-GFP (Green) were stained with mitotracker568 (red) to detect mitochondria and TOTO3 (red) to stain nuclear chromatin. (**D**) Jig protein binds to nuclear chromatin. Salivary glands were dissected from third instar larvae expressing Jig-GFP, squashed and stained with anti-GFP antibody (Red); DNA was detected using TOTO3 dye (Green). N – nucleus. Scale bars, 15 μm.

To monitor the subcellular localization of Jig protein, we expressed the UAS-Jig-GFP transgenic reporter using ubiquitous GAL4 driver. An immunoblot analysis using a GFP antibody (αGFP) demonstrated that the transgene produces a single 46-kDa protein (Figure 1G). This expression is well tolerated by animals and has no effects on *Drosophila* development, viability, fertility or health. Confocal microscopy of dissected tissues of 12hrs third instar larvae expressing recombinant Jig-GFP identified Jig as a protein localized to both nucleus and cytoplasm. In the nucleus, one fraction is bound to chromatin and the other enriched in nucleoli (Figure 2A and Supplemental Figure S3). Coexpression of Jig-GFP with mitochondrial protein TIM17b-DsRed (15) (Figure 2B) and co-staining with mitotracker reagent (Figure 2C) showed that those cytoplasmic organelles where Jig localizes are mitochondria. The analysis of Jig protein binding to chromatin using immunostaining of larval polytene chromosomes squash demonstrated that Jig binds to approximately 200 loci in the euchromatic portion of *Drosophila* genome (Figure 2D).

**Figure 3.**
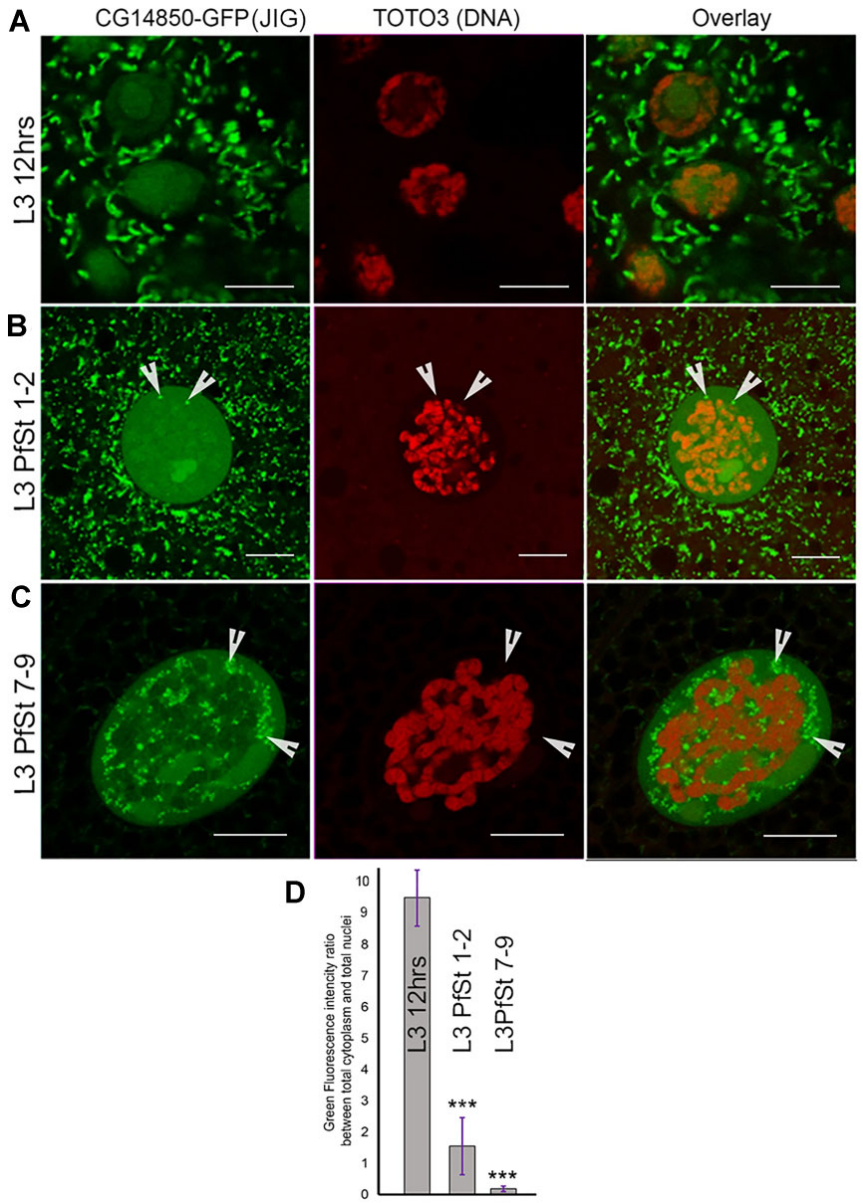
Jig protein localization changes during late third instar larval stages. (**A**) Jig-GFP protein localizes to mitochondria and in nuclear chromatin during early third instar larvae stage. (**B**) Jig-GFP starts to accumulate in extrachromosomal nuclear particles (arrowheads) by the puff stage in 1–2 third instar larvae. (**C**) Jig-GFP is mostly lost from mitochondria and chromatin and enriched in extrachromosomal nuclear particles (arrowheads) by puff stages 7–9 (37). (**D**) Jig protein relocalizes from mitochondria to nucleoplasm during third instar larvae development. Quantification of the ratio between the intensity of total cytoplasm and total nucleoplasm fluorescence was calculated for the L3 12 h, L3 PfSt 1–2 and L3 PfSt 7–9 samples. Experiments were performed in 10 biological replicates with mean ± the standard error of the mean graphed for the distinct L3 stages. ‘N’ marks nuclei. *** *P*-value ≤ 0.05. Scale bars, 15 μm.

We found that intracellular distribution of Jig protein changes during third instar larval development (Figure 3). Intrinsic Jig is only expressed from L3 12 h stage to prepupae stage, so we tested only this period. Based on quantification of confocal microscopy pictures, by stage L3 12 h, 73% of the total Jig-GFP had accumulated in mitochondria, while 27% had bound to nuclear chromatin and nucleoli (Figure 3A). By L3 PfSt 1–2, the distribution had changed to 56% in mitochondria and 44% in nuclei (Figure 3B). Aside from binding to chromatin and nucleoli, at this later stage in development, Jig also accumulates in extra-chromosomal bodies (Figure 3B, arrowheads). By stage L3 PfSt 7–9, 94% of Jig-GFP has already translocated to nuclei, has been excluded from chromatin and nucleoli, and is now primarily localized to extra-chromosomal bodies (Figure 3C). Analysis of confocal microscopy images confirmed the progressive re-localization of Jig protein from mitochondria into nucleoplasm during third instar larval development (Figure 3D). Taken together, these data strongly suggest that Jig protein may play some role in nuclear-mitochondrial communication and likely coordinates nuclear and mitochondrial functions.

### Jig protein is required for mitochondria

**Figure 4.**
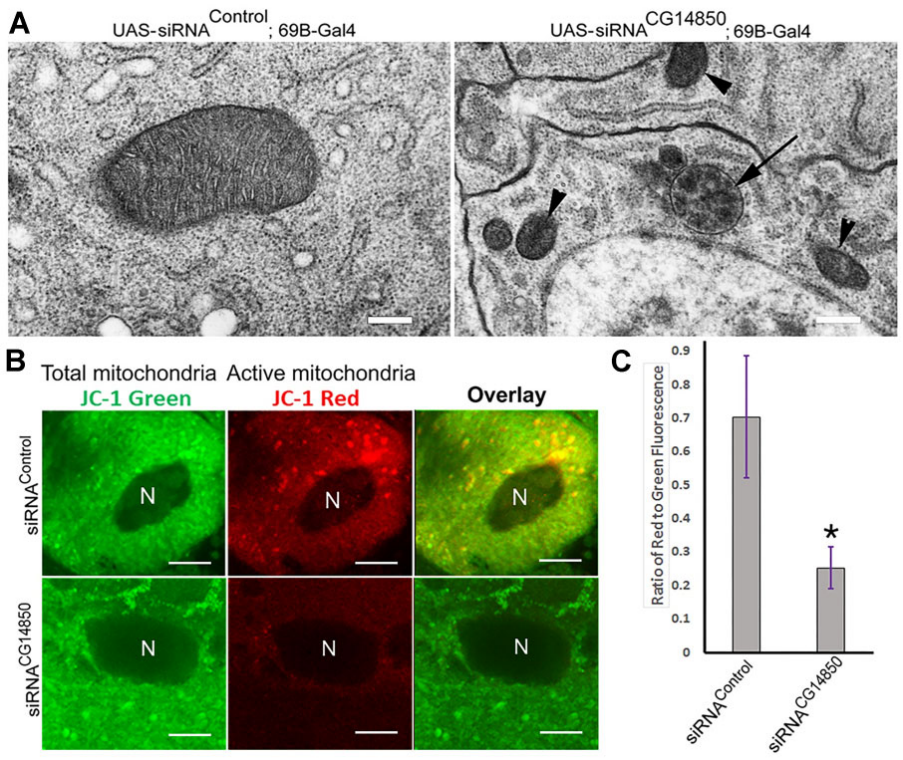
Jig protein is required for mitochondria functions and morphology. (**A**) Mitochondrial morphology is affected in Jig knockdowns. siRNA transgenic constructs against Jig (right panel) or control siRNA (left panel) were expressed using 69B-Gal4 drivers in wild-type flies. Salivary glands were dissected and subjected to transmission electron microscopy analysis. Arrowheads indicate abnormally small mitochondria in *Jig* knockdowns. The arrow indicates abnormal mitochondria with cristae inside. The white bar corresponds to 2 μm. (**B**, **C**) Mutating Jig disrupts mitochondrial function based on the JC-1 mitochondrial membrane potential assay. siRNA transgenic constructs against Jig or control siRNA were expressed using 69B-Gal4 drivers in wild-type flies. Salivary glands were dissected and stained alive with JC-1 which stains all mitochondria green, while staining the physiologically active mitochondria red (B); then the ratio (C) between the intensity of green and red fluorescence was calculated for the control and experimental samples. Experiments were performed in three biological replicates with the mean and standard error of the mean graphed. ‘N’ marks nuclei. * *P*-value ≤ 0.05. Scale bars, 15 μm.

To test whether Jig plays any role in mitochondrial stability and/or survival, we first analyzed if mitochondrial morphology is affected in absence of Jig using transmission electron microscopy (Figure 4A). Tissues from the animals expressing control siRNA and siRNA against Jig were dissected from L3 12hrs stage larvae and compared in respect to mitochondrial morphology. Strikingly, typical mitochondria (Figure 4A, left) were scarce in Jig knockdowns (Figure 4A, right). Instead, we observed a large number of small mitochondria (Figure 4A, arrowheads), as well as mitochondria with an abnormal phenotype that retained residual cristae inside (Figure 4A, arrow). Although the morphology of mitochondria is significantly disrupted the functions of mitochondria seems to be not affected (Supplemental Figure S4).

To test whether Jig-knockdown mitochondria are still metabolically active, we employed the JC-1 mitochondrial membrane potential assay (29), which is used to quantify the fraction of active mitochondria in the cell using confocal microscopy. JC-1 is a fluorescent dye that exists as green-emitting monomers in solution, but these monomers can reversibly aggregate in mitochondria with high membrane potential, forming red-emitting complexes and thus highlighting healthy organelles. Data presented in Figure 4B, C clearly demonstrate that knocking Jig down severely diminishes the active mitochondrial fraction. This last observation strongly supports our hypothesis that Jig plays an important role in supporting normal mitochondrial metabolism.

### Jig protein is a component of CREB protein complex

**Figure 5.**
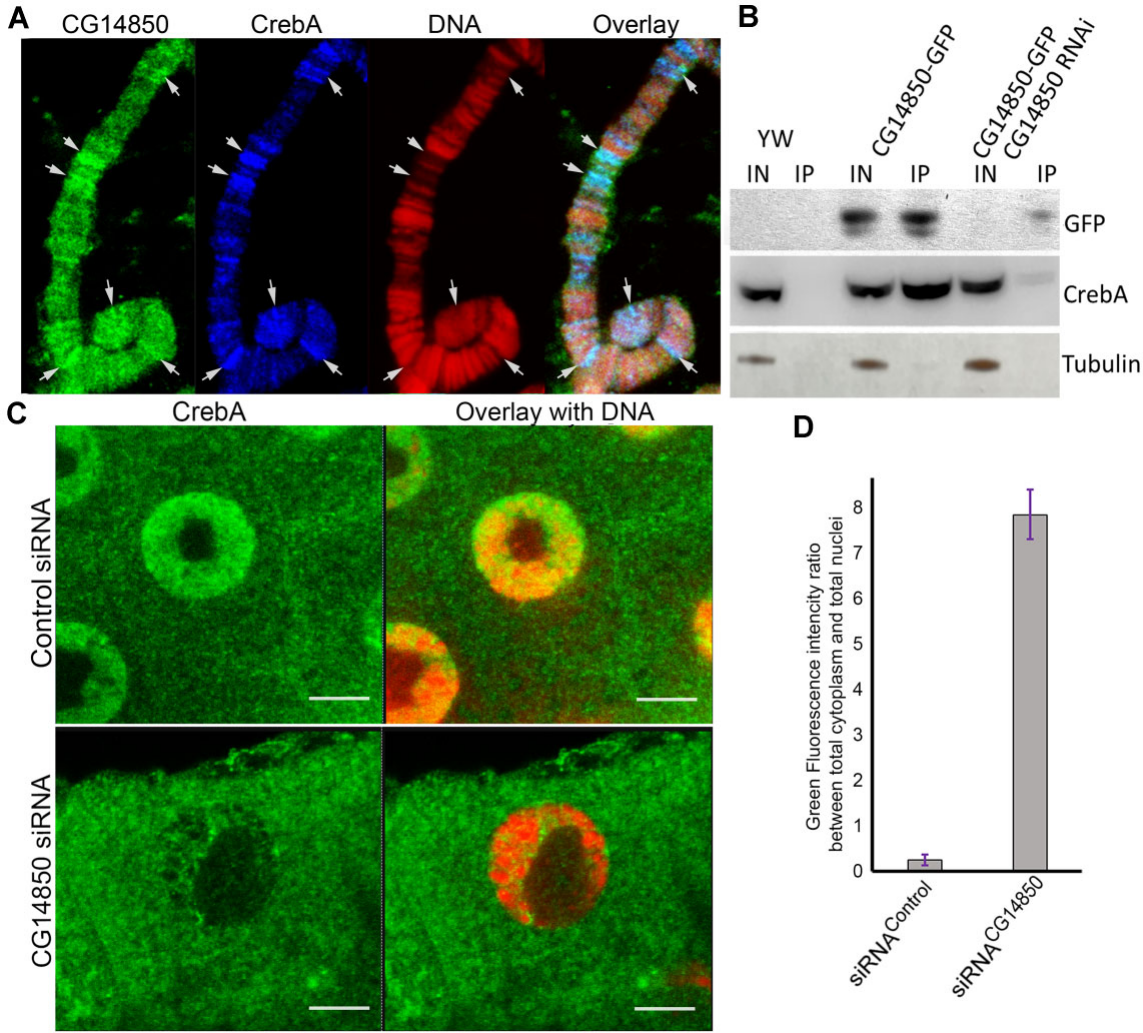
Jig protein controls CrebA complex localization in nuclei. (**A**) Jig and CrebA proteins are colocalized in chromatin. Salivary glands of *Drosophila* larvae expressing Jig-GFP were dissected from third instar larvae, squashed and stained with monoclonal anti-GFP (green) and polyclonal anti-CrebA (red) antibodies; DNA was detected using TOTO3 (blue). Arrows indicate sites of obvious colocalization of Jig and CrebA in polytene chromosomes. (**B**) Jig protein interacts with CrebA in *Drosophila*. Immunoprecipitation assays using monoclonal anti-GFP antibody. *Drosophila* stocks expressing Jig-GFP or coexpressing Jig-GFP and Jig siRNA were used. Wild-type (WT) *Drosophila* stock was used as a control. To detect protein on Western blots, the following antibodies were used: rabbit anti-CrebA; rabbit anti-GFP (to detect Jig-GFP); rabbit anti-Tubulin. (**C**, **D**) Jig protein is required for CrebA complex delivery to nuclei. siRNA transgenic constructs against Jig (right panel) or control siRNA (left panel) were expressed using 69B-Gal4 drivers in wild-type flies. Salivary glands were dissected from third instar larvae L3 12 h stage and subjected to immunostaining using anti-CrebA antibody (green). DNA was stained using TOTO3 dye (red). The ratio (D) between the green fluorescence intensity in total cytoplasm and total nuclei was calculated for the control and experimental samples. Experiments were performed in three biological replicates. 5–10 cells were analyzed in each experiment. *** *P*-value ≤ 0.05. Scale bars, 15 μm.

CREB protein complex plays important roles in mitochondrial and nuclear transcription (11,41). Two independent groups reported that *Drosophila* CrebA protein interacted with Jig in yeast two-hybrid (38,39). To confirm the functional interaction of Jig with CREB protein complex in the cell, we first tested if these proteins colocalized *in vivo* using immunostaining. First, we confirmed that *Drosophila* CrebA protein localizes to the nuclei and mitochondria (Supplemental Figure S5). To determine if Jig and CrebA colocalize in *Drosophila* chromatin we performed immunostaining of larval polytene chromosomes squash for CrebA and Jig-GFP. Notably, almost 100% of CrebA-positive sites were also occupied by Jig (Figure 5A) (Supplemental Figures S6 and S7). Co-immunoprecipitation shows that Jig directly interacts with CrebA protein (Figure 5B). Nuclear localization of CrebA protein, which is normally detected in wild-type *Drosophila* tissues (Supplemental Figure S5), is severely diminished in Jig knockdowns (Figure 5C, D). In addition, the localization of CerbA protein at the chromatin is severely impaired in Jig knockdown (Supplemental Figure S8). This data strongly suggests that Jig is required for CrebA localization at the nucleoplasm.

### Jig protein together with CrebA binds promoters in nuclear and mitochondrial genomes

**Figure 6.**
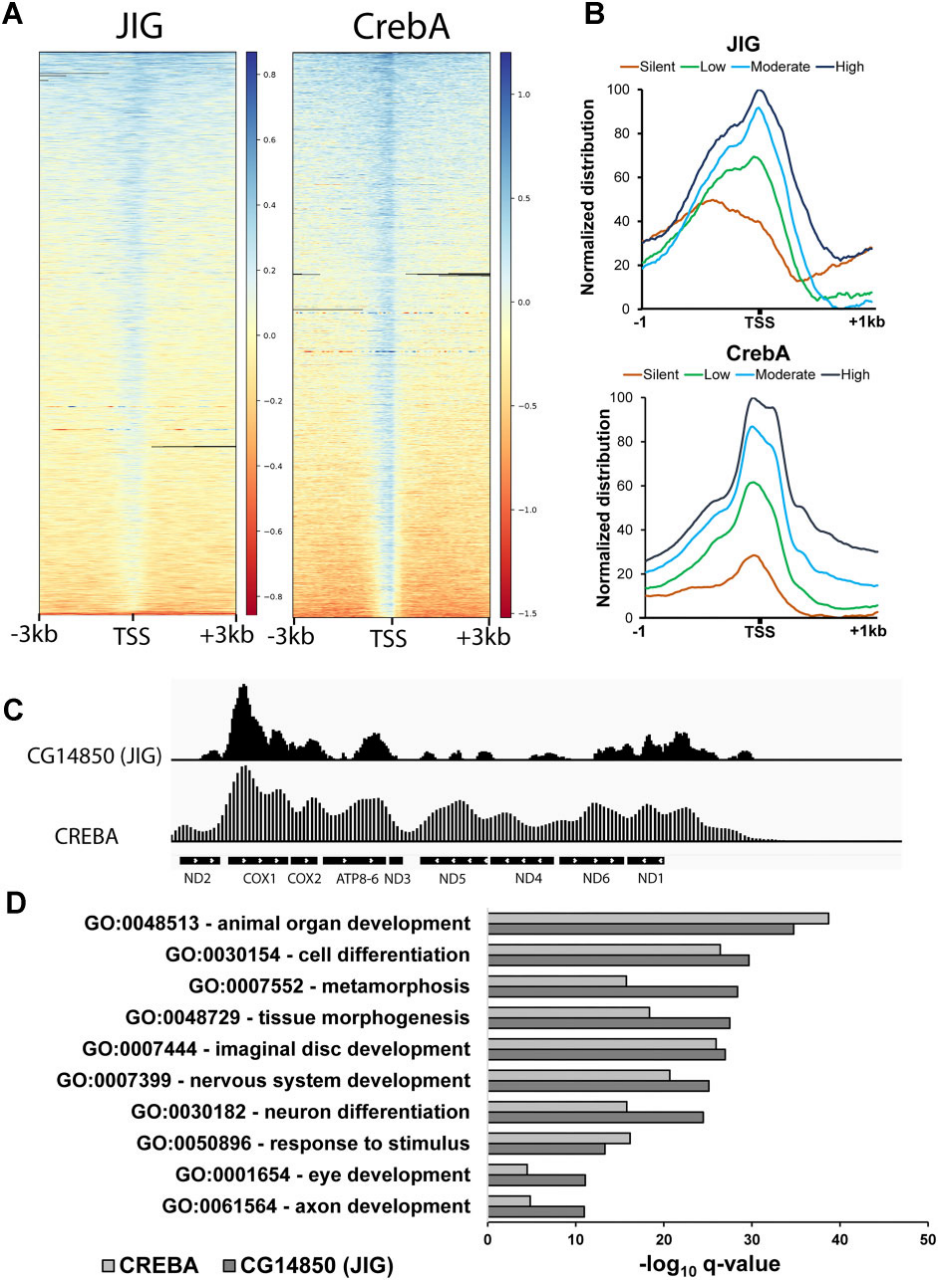
Jig and CrebA are colocalized in the mitochondrial and nuclear genome at the TSS of active genes. (**A**) Heatmap showing ChIP-seq signals of Jig and CrebA. Jig and CrebA are mostly bound to the TSS across the genome. (**B**) Metagene plot of ChIP-seq signals of Jig and CrebA across promoters based on expression quartiles from WT third-instar larvae RNA-seq data (0–25% = silent; 25–50% = low; 50–75% = moderate; 75–100% = High). (**C**) pyGenomeTracks showing the distribution of Jig and CrebA across the mitochondrial genome. (**D**) Most overrepresented gene ontology terms among Jig or CrebA target genes. X-axis corresponds to the FDR-adjusted *P*-value (*q*-value) for each term. Overrepresented gene ontology terms are involved in development, morphology and differentiation.

To determine the exact genomic distribution of Jig protein in *Drosophila* larvae, we performed ChIP-seq assays with anti-GFP antibody. We performed ChIP-seq with wild-type *Drosophila* as a background control. Analysis of Jig occupancy in the nuclear genome identified two groups of Jig binding sites: unique and repetitive genomic sequences (transposons), suggesting that Jig plays a role in the transcriptional regulation of both unique loci and repetitive DNA. We identified 1476 Jig binding sites, among which 1469 are in the nucleus and 7 in the mitochondria. We identified 461 sites that Jig bound to be in repetitive regions. Similar to CrebA, Jig bounds mostly to the promoter region near the transcriptional start sites (TSSs) (Figure 6A, Supplemental Figure S9) suggesting that Jig, together with CrebA, is involved in regulation of gene expression. Jig and CrebA binding profile on genomic regions ranging from highly active to silent genes is also the same and they both bound mostly to active genes (Figure 6B). We found Jig and CrebA proteins to be broadly bound along the mitochondrial genome and present an identical binding profile (Figure 6C). Finally, mutating JIG disrupts the expression of JIG-occupied loci (Supplemental Figure S10). These results suggest that Jig and CrebA bind together to the promoter region of common nuclear loci and to the mitochondrial genome.

Next, we investigated the functions of Jig and CrebA target genes. Jig bound to within –5 and +5 kb of 966 genes. Seventy percent (678) of these genes are active during the first 12 h of third instar larval development (37). Interestingly, the Human orthologs of 74.5% of Jig target genes are reported to be CREB targets (27,28). To determine if Jig and CrebA target genes share similar functions we compared gene ontology (GO) of Jig and CrebA target genes. We found that the most enriched functions are common for both Jig and CrebA targets (Figure 6D). These functions belong to three main categories, development, morphogenesis and differentiation, suggesting that Jig and CrebA regulate the expression of developmental genes (25). Finally, several CrebA binding motifs have been identified that all share a similar central sequence of ‘CCACGTC’ (34,40,42). Notably, this motif is present at 69% of Jig-occupied promoters. Collectively, our findings suggest that Jig together with CrebA play a major role in *Drosophila* development through gene transcriptional regulation in both nuclear and mitochondrial genome.

## DISCUSSION

In this study, we demonstrated that a protein with previously unknown function, which we termed as Jig, localizes to both mitochondria and nucleus, a rare phenomenon demonstrated by only a handful of proteins. As stated before, this type of dual-localized protein is often involved in the communication between mitochondria and nucleus, which is essential for cell survival. Mitochondrion sends signals to the nucleus to transcribe genes that will help to carry out its function and/or to help relieve stress. Similarly, nucleus can also send signals to mitochondria to transcribe genes that will help bring the cell to homeostasis, as needed (43,44). The importance of this nuclear-mitochondrial communication has led many to investigate proteins localized in both mitochondria and nucleus. However, this communication pathway is still not fully understood. When we identified the Jig protein and determined that it localized to both nuclear and mitochondrial compartments, it prompted a thorough investigation into this protein's potential function in mito-nuclear communication. Through ChIP sequencing and chromosome squash imaging, we showed that this protein not only has dual localization but also binds to the genomes of both nuclear and mitochondrial compartments.

The unique temporal and spatial expression pattern of Jig protein localization is specific to the third larval stage of *Drosophila melanogaster* development. Jig moves from mitochondria to nucleus from early to late third instar larval stage. The distribution of this protein is highest in the mitochondria at the earlier third larval stage and highest in the nucleus near the end of the third larval stage (Figure 3). Jig is vital for *Drosophila* development and its knockdown causes developmental arrest in *Drosophila* at third larval stage (Figure 1H). This developmental stage (larva L3 12 h) at which this arrest occurs coincides with the exclusive developmental stage at which Jig is expressed in wild type *Drosophila* (Figure 1C). Knocking down Jig disrupts normal mitochondrial shapes and sizes, leading to mitochondria with decreased membrane potential (Figure 4). We also showed that Jig does not function alone. It binds to *Drosophila* CREB. This protein has been a subject of immense study in mammals for understanding its role in nuclear and mitochondrial communication. Ever since the discovery of retrograde response genes (RTGs) in yeast that mediate mitochondria signaling to the nucleus, thus establishing a retrograde communication pathway with it, researchers have been trying to find equivalent genes in mammals. Instead, in mammals, they found that this function is served by several genes that establish a retrograde communication signaling pathway between mitochondria and the nucleus. CREB is one of these proteins (12).

Under steady state condition, CREB translocates to mitochondria (45,46). When a lot of changes are happening inside a cell, like during development, mitochondria send signal to nucleus through translocation of nuclear transcription factors like CREB to the nucleus to regulate genes (47). Here we showed that Jig protein binds to CREB and colocalizes with CREB in the nuclear chromatin, whereas Jig knockdown disrupts CREB localization into the nucleus (Figure 5C, D). When mitochondria need to communicate with the nucleus, the Jig protein ‘hooks’ CREB and facilitates its trafficking to the nucleus where CREB can bind to genes to regulate nuclear/mitochondrial functions. Thus, any dysfunction in mitochondria will lead to retrograde signaling carried by proteins like CREB to help return cells to homeostasis. Therefore, it is not surprising that knockdown of Jig prevents CREB accumulation in the nucleus and causes mitochondria to lose their functionality and become smaller and less active. These compromised mitochondria are energetically impaired by the loss of nuclear communication. This leads to eventual arrest of growth and lethality in Jig knockdown *Drosophila*.

Both Jig and CrebA bound to almost the whole mitochondrial genome in exactly the same pattern, suggesting a Jig-CrebA dependent regulation of these genes. These genes code for subunits of Oxidative phosphorylation pathway proteins (48). This explains why knockdown of Jig causes morphological and functional changes in mitochondria (Figure 4A–C). Interestingly, despite the morphology of mitochondria that is significantly disrupted, the functions of mitochondria do not seem affected (Supplemental Figure S4). This result could be explained by a global downregulation of metabolism during the third instar larvae to prepare metamorphosis (49–51), that could mask the effect of Jig on mitochondrial metabolism. Furthermore, we showed that Jig and CrebA bind together mostly to active genes (Figure 6B). These genes are mainly involved in developmental processes (Figure 6D). Developmental arrest during third instar larval stage that we observed when Jig function is disrupted could be due CrebA no longer being transported to the nucleus, leading to a misregulation of the expression of these developmental genes (Figures 1H, 6D). In parallel, we identified loci where Jig binds, while CrebA is absent. This result could reveal a second role of Jig in the regulation of gene expression that is CrebA-independent.

Even though it performs a vital function, Jig is only present in *Drosophila* during a specific developmental stage. We predict that this vital function is carried out by its paralogues (Figure 1F) when Jig is no longer present. Paralogues, such as CG11300, CG14852 and CG12491, are expressed in either the first larval or the embryonic stage too, potentially carrying out a function similar to that of Jig protein (14). Jig protein has a conserved region of 5 cysteines that are also present in a group of its paralogues: CG14851, CG13135 and CG8087 (Supplemental Figure S2). Interestingly, these are not the only proteins with these conserved cysteine groups since they are also found in the Arabidopsis plant species. This group of proteins is named as Plasmodesmata callose binding proteins (PDCBs) because they function in cell-cell trafficking by anchoring to the plasma membrane using the part of the structure that contains the 5-cysteine group (52). One possible interpretation of the structure/function relationship in the Jig protein is that the cysteine-based motif helps in anchoring CREB to DNA. Along with that feature, Jig has a strong positively charged Arginine region at its C-terminal end which helps it to localize to the nucleus. It is well known that CREB forms a complex with other proteins like CREB binding protein, ATF1, in the region of the gene it is going to transcribe (53). Because of its structure, our model suggests that Jig plays an anchoring role for this complex on the gene promoter.

Jig interacts with other TFs, such as LOLA and XBP1. LOLA is important for neural development of *Drosophila* during the embryonic stage (54). Its knockdown leads to problems with axonal growth causing lethality. XBP1 is involved in the unfolded protein response (UPR) pathway (55). It transcribes genes that can degrade and/or fold the unfolded proteins in endoplasmic reticulum. Jig also interacts with SKPA, another protein involved in protein degradation. SKPA degrades ubiquitin-tagged proteins (56). Interestingly, SKPA knockdown also causes lethality owing to motor dysfunction. The disappearance of Jig after third larval stage, together with its interaction with XBP1 and SKPA, suggests its potential degradation via the UPR pathway, or through ubiquitination and degradation by SKPA, after Jig is done delivering CREB to the nucleus and helping it to transcribe the necessary genes. Other interactors of Jig, such as ODA and DIDUM, are also vital for *Drosophila* development and, similar to Jig knockdown, knocking down any of them results in lethality before the end of larval stage (31). Interestingly, Jig interactors show a bias towards neural development. So, it is possible that Jig, along with its function to traffic and anchor CREB to nucleus, also functions in neural development pathways through its connection with its interactors and its critical role in metabolic homeostasis. This could be an interesting area of study for future research on Jig.

## DATA AVAILABILITY

Mutant strains and transgenic stocks are available upon request. The authors state that all data necessary to confirm the conclusions presented in the article are represented fully within the article. High throughput sequencing has been deposited in GEO under accession number GSE193786 (https://www.ncbi.nlm.nih.gov/geo/).

## Supplementary Material

gkad343_Supplemental_File
