# Supplementary material for: The Drosophila gene encoding JIG protein (CG14850) is critical for CrebA nuclear trafficking during development
Source: Nucleic Acids Res. 2023 May 5;51(11):5647–60. doi: 10.1093/nar/gkad343 (PMC10287909; doi:10.1093/nar/gkad343)
Supplement: gkad343_Supplemental_File [file gkad343_supplemental_file.pdf]

SUPPLEMENTAL FIGURES

The Drosophila gene encoding JIG protein (CG14850) is critical for CrebA nuclear trafficking during development

Sayem H. Bhuiyan, Guillaume Bordet, Gbolahan Bamgbose and Alexei V. Tulin

University of North Dakota, Grand Forks, ND

Address correspondence to:

Alexei V. Tulin, Ph.D.

Department of Biomedical Sciences

School of Medicine and Health Sciences

University of North Dakota

501 North Columbia Road, Stop 9061

Grand Forks, ND 58202

Phone: 1 701 777 4922; FAX: 1 701 777 2054

E-mail: Alexei.Tulin@und.edu

Running head: Jig mediates nuclear-mitochondrial communication

Keywords: CG14850, CREB, Mitochondria, Nuclear chromatin, *Drosophila*

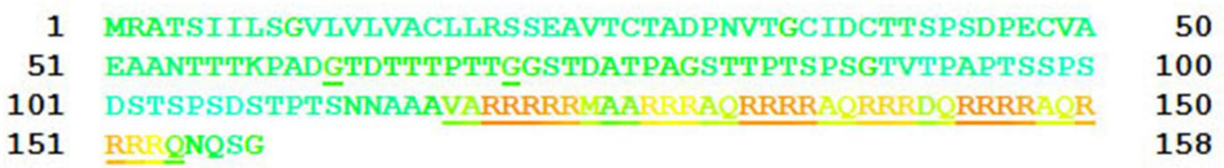

Supplemental Figure S1. Nuclear Localization Signal (NLS) is identified in Jig protein sequence using the NucPred software. It shows that Jig has a strong positively-charged C-terminal end owing to repeats of many positively-charged amino acids.

A. CG14850(Jig)/CG14851/CG8087/CG13135:

```
CG14850      MRATSIILSGVLVLVACLLRSSE-AVTCTADPNVTGCIDCTTSPSDPE--CVAEAA-NTT-TK-----P
CG14851      MRAATIIF-AILVLAACLLRSSE-AVTCTADPTVTGCINCTTNPTDSE--CVAEAAAA-TSTTTTVA-P
CG8087       MKATTILA-VVSVLTACLLRSSE-AVTCTADATVTGCIDCTTNPTDSE--CVAEAAADTTSTT--VATP
CG13135      MRKSLLIVGSLLVTIFLAHLPVGLAVSCADDPTDTACIDCTDTANAAEADC----------TTTTAA P

CG14850      ADGTDTTTPTTGGSTDATPAGSTTPTSPSGTV----------------TPAP TSSPSDSTSPSDSTPT
CG14851      ATSTVATTTSATATTTAASSTNTSSGRRKIVRITNLRYT NVRRIRVN---R----NG-SG----ST-T
CG8087       -TTT-ATTT--TAPTT--SSG-TGTGSRKIVRVSNLRYSVN-RRIRINTTARSTSSNGRSGRR--STNT
CG13135      EVTTAAAEVTTAASADGETTTAAASATDTTTASSGGSGKKRVRRTFRR---KV------------SR-P

CG14850      SNNAAAVARRRRRMAARRRAQRRRRAQRRRDQRRRRAQRRRRQNQSG
CG14851      VRN-----RRRRNN-SRRVNVRRANGNV---------------IVVG
CG8087       NRN-----RRRRNNNARQGNSRSSRGNVR--------------VVVG
CG13135      -RK----IKKRRSNIKKRRSNRGR--------------NNRRSQNSG
```

B. CG14850(Jig)/CG14852:

```
CG14850      MRATSIILS-GVLVLVACLLRSSEAVTCTADPNV--TGCIDCTTSPSDPECVAEAANTTTKPADGTDTT
CG14852      MRTTTLLLSLGLLVL--C--FSSYSF-AEDDPTDGSTTPTDGSTTPTDGSTT--PTDGSTTPTDGSNT-

CG14850      TPTTGGSTDATPA-GSTTPTSPSGTVTPA-PTSSPSD-STSPSDSTPTS--------------------
CG14852      -PTDGSTT---PTDGSTTPTDGS--TTPTDGSTTPTDGSTSPSTS-PSTGDNTSPSTGSPDSTPDSGSG

CG14850      -----NNAAAVARRRRRMAARRRAQ--RRRRAQ--RRRDQRRRRAQRRRRQNQSG
CG14852      SNNSGNNKRNN-RRRRRQ----RAQRARRRRAQQARRRRNRRNNRNNRLSRTNSI
```

C. CG14850(Jig)/CG11300:

```
CG14850      MRATSIILSGVLVLVACLLRSSEAVTCTADPNVTGCIDCTTSPSDPECVAEAANTTTKPADGTDT---T
CG11300      MRCQFVIAFGLLALIA------------------TAYADSPPAAGSPPASSPPAGTPTSPPPATGTPPSP

CG14850      TPTTGGSTDATPAGST---------TPTSPSGTVTPAPTSSPSDSTSPSDSTPTSNNAAAVARRRRRMA
CG11300      SPATGTPPSASPAAGTPTSPTPATGTPSSPATPDAPASSTSPATPTSPSDS--GSSSSQEVIRLRRRLR

CG14850      ARRRAQRRRRAQRRRDQRR---------RRAQRRRR
CG11300      RLRRQLRRERRQANQSNQNGGGGQGRVVRRVHRHRR
```

D. CG14850(Jig)/CG12491:

```
CG14850      MRATSIILSGVLVLVACLLRSSEAVTCTADPNVTGCIDCTTSPSDPECVAEAANTTTKPADGTDTTTPT
CG12491      MRPEFVLAFGLVVLVATVYGGTDSSSSDS--------SSSTSPTSNSSTPSTSSSSSTPSSSSSTSTPS

CG14850      TGGST---DATP--AGSTTPTSPSGTVTPAPTSSPSDSTSPSDSTPTSNNAAAVARRRRRMAARRRAQR
CG12491      SNSTTSTSSSTPSSSSSTSPTSSTSSTT-ATTTAPSTSSDTSSSS-TSSDSEEVDRLRRRL---RRLRR

CG14850      RRRAQRRRDQRRRRAQRRRRQNQSG
CG12491      LRRQERRQEIRRERQQERRQQSRAG
```

E. CG14850(Jig)/CG14421:

```
CG14850      ILSGVLVLVACLLRSSEAVTCTADP-------------------NVTGCIDCTTS-PSDPECVAEAANT
CG14421      LLKGLLI-VACL-----AAFASAKPQNFQIFGRQRDEPAAQGRLSATQLTDLLNSLKGSSSSTTAAPTT

CG14850      TTKPADGTDTTTPTTGGSTDATPAGSTTP--TSPSGTVTPAPTSSPSDSTSPSDSTPT-----------
CG14421      TTLPSTSTVTTSPTGPTTSTGTSTTTTTPTTTSPTGTTT-ARQLEPLDDQEDDDEEEQQLGHHQRFQLE

CG14850      ---------SNNAAAVARRRRRMAARR-------------RAQRRRRAQ----RRRDQRRRRAQRRRR-
CG14421      DQNDDEEEHTQVARSQANRRRQVNARRQRQRLQQKYRRRQQQQRRRRQQQQQKRRQQQKRRQQQRRRRQ

CG14850      QNQ
CG14421      QNQ
```

F. CG14850(Jig)/CG16953:

```
CG14850      PECVAEAANTTTKPADGTDTTTPTTGGSTDATPAGSTTPTSPSGTVTPAPTSSPSDSTSPSDSTPTSNN
CG16953      PEPTPSTTTTTT-------TTTTTTTTTTTTPATTTTSTTPATTTTPKTTTS-------STSTTTTTT

CG14850      AAAVARRRR
CG16953      PKPPARSKR
```

G. CG14850(Jig)/CG4297:

```
CG14850      SPSDPECVAEAANTTTKPADGTDTTTPTTGGSTDATPAGSTTPTSPSGTVTPAPTSSPSDSTSPSDSTP
CG4297       SSSGP---GSSSSGSSSSGYGSATTTPTSGGHYDNSP--TSMPMATIATVAP----FYSEALTSLDA--

CG14850      TSNNAAAVARRRRRMAARRRAQRRRRAQRRRDQRRRRAQRRRRQNQS
CG4297       --------------------------------QQRQQQQQQQQQNPS
```

Supplemental Figure S2. Paralogs of JIG protein in D. melanogaster genome. Alignments of JIG paralogues from *Drosophila melanogaster* genome. **A.** CG14850(Jig), CG14851, CG8087 and CG13135; **B.** CG14850(Jig) and CG14852; **C.** CG14850(Jig) and CG11300; **D.** CG14850(Jig) and

CG12491; **E.** CG14850(Jig) and CG14421; **F.** CG14850(Jig) and CG16953; **G.** CG14850(Jig) and CG4297

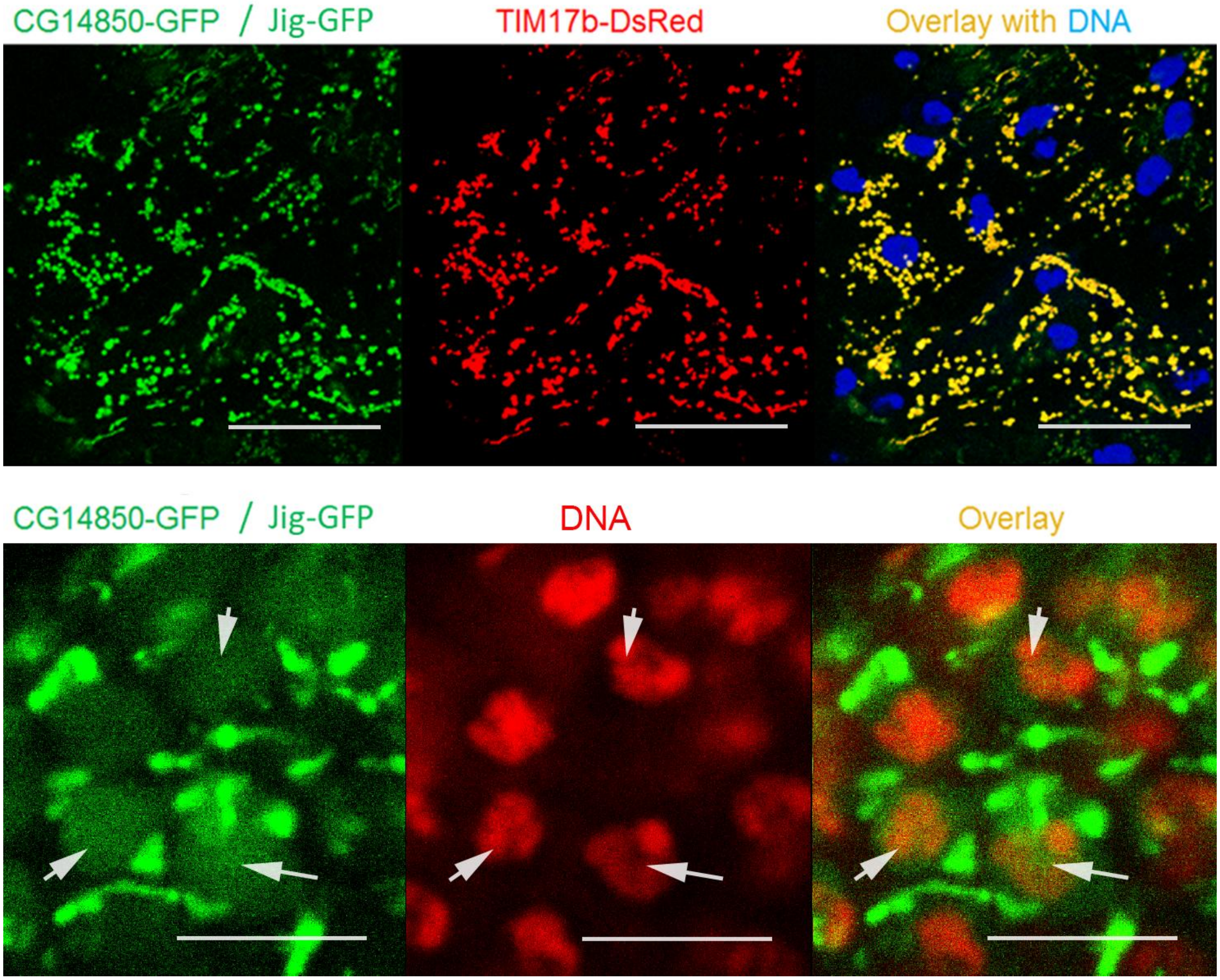

Supplemental Figure S3. Jig protein is localized to mitochondria (top) and nuclei (bottom) in diploid tissues. Top: Third instar larvae imaginal discs (eye) tissues expressing Jig-GFP (Green) and mitochondrial protein TIM17B-DsRed [20] (Red) were stained with TOTO3 (Blue) to stain nuclear chromatin. Scale bars, 15μm. **Bottom:** Third instar larvae imaginal discs (eye) tissues expressing Jig-GFP (Green) were stained with TOTO3 (Red) to stain nuclear chromatin. Arrows are showing localization of Jig protein in nuclei. Scale bars, 5μm.

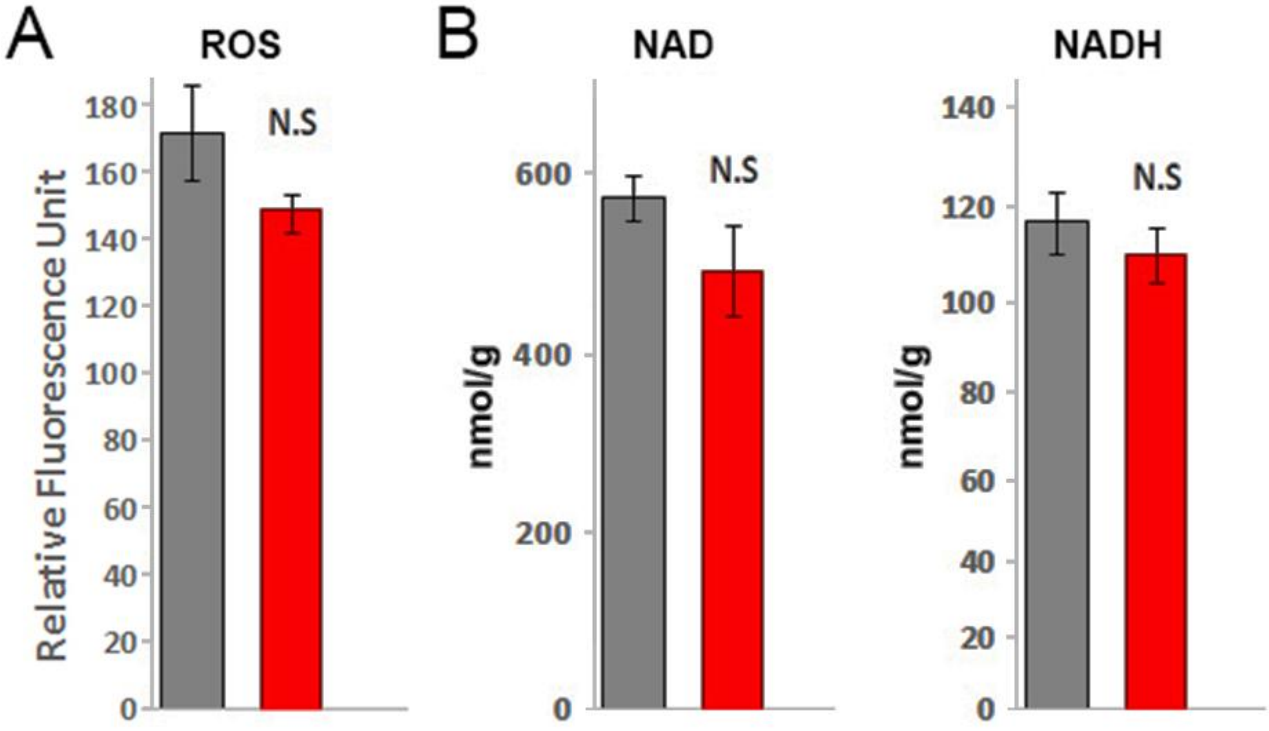

Supplemental Figure S4. A. The assay shows no excess in ROS production due to JIG knockdown. The Y axis represents the Relative Fluorescence Unit (RFU) of H2DCF dye. More RFU represents more ROS production. There is no significant ROS production due to Jig knockdown compared to control. The experiment was done in triplicates and p value was found to be > 0.05 through unpaired T-test. **B.** NAD and NADH Levels in wild type (siRNA control (grey)) and in JIG KD (Red) N.S means not significant.

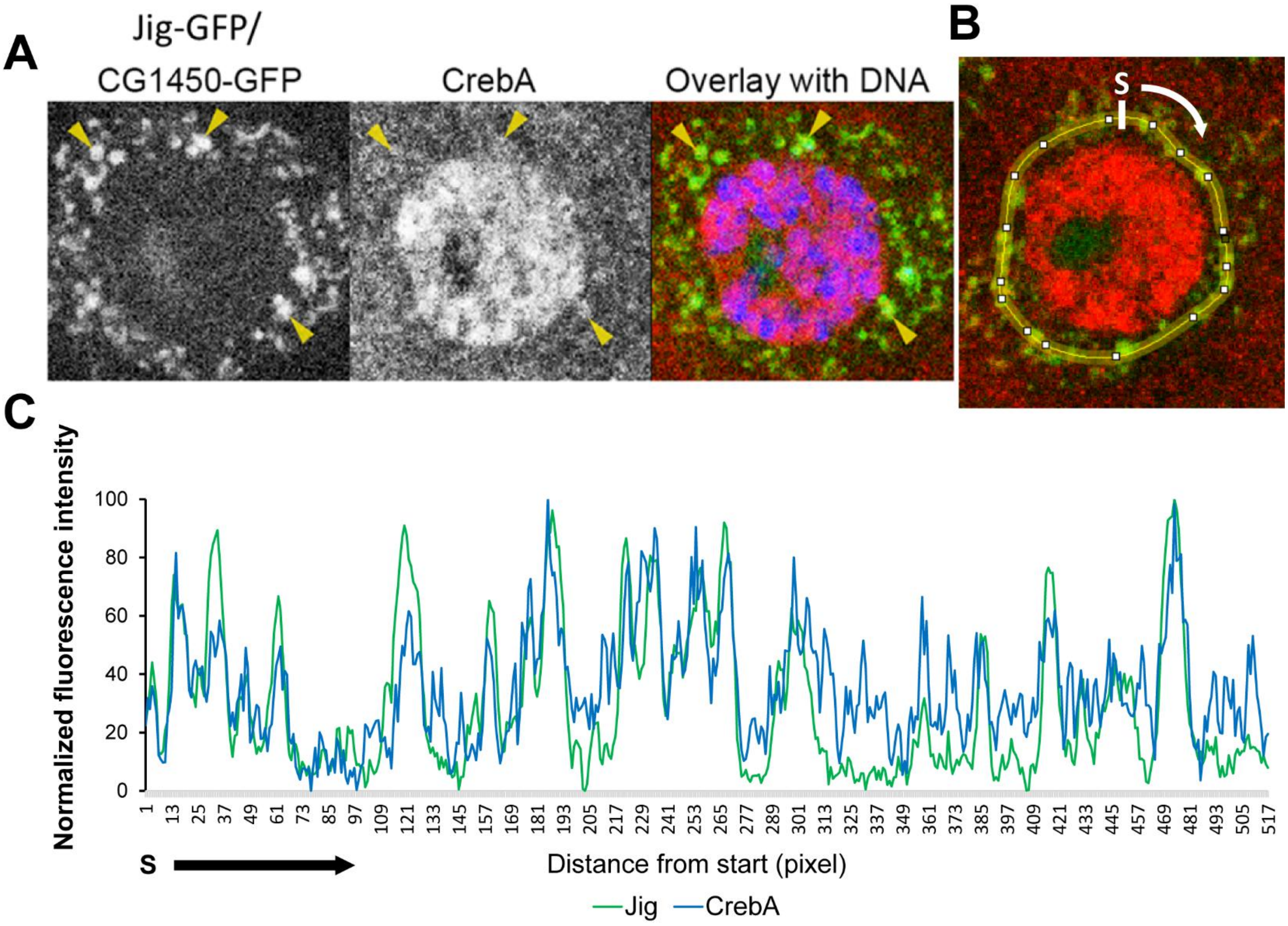

Supplemental Figure S5. Jig and CrebA are colocalized in mitochondria (arrows). A. Immunostaining of *Drosophila* 3rd instar larvae L3 12hrs stage salivary glands using monoclonal anti-GFP (green); polyclonal anti-CrebA (red) antibodies: a single cell is shown; DNA is blue. Arrowheads show mitochondria. Scale bars, 15µm. **B-C.** Analysis of the distribution of CrebA and Jig fluorescence intensity. **B.** ROI used to measure CrebA and Jig distribution, starting from the "S" marker and rotating clockwise. **C.** Distribution of CrebA and Jig fluorescence intensity. X-axis corresponds to the distance from start ("S" marker on panel B) in pixel. Signal is normalized (0-100%) for a better visualization.

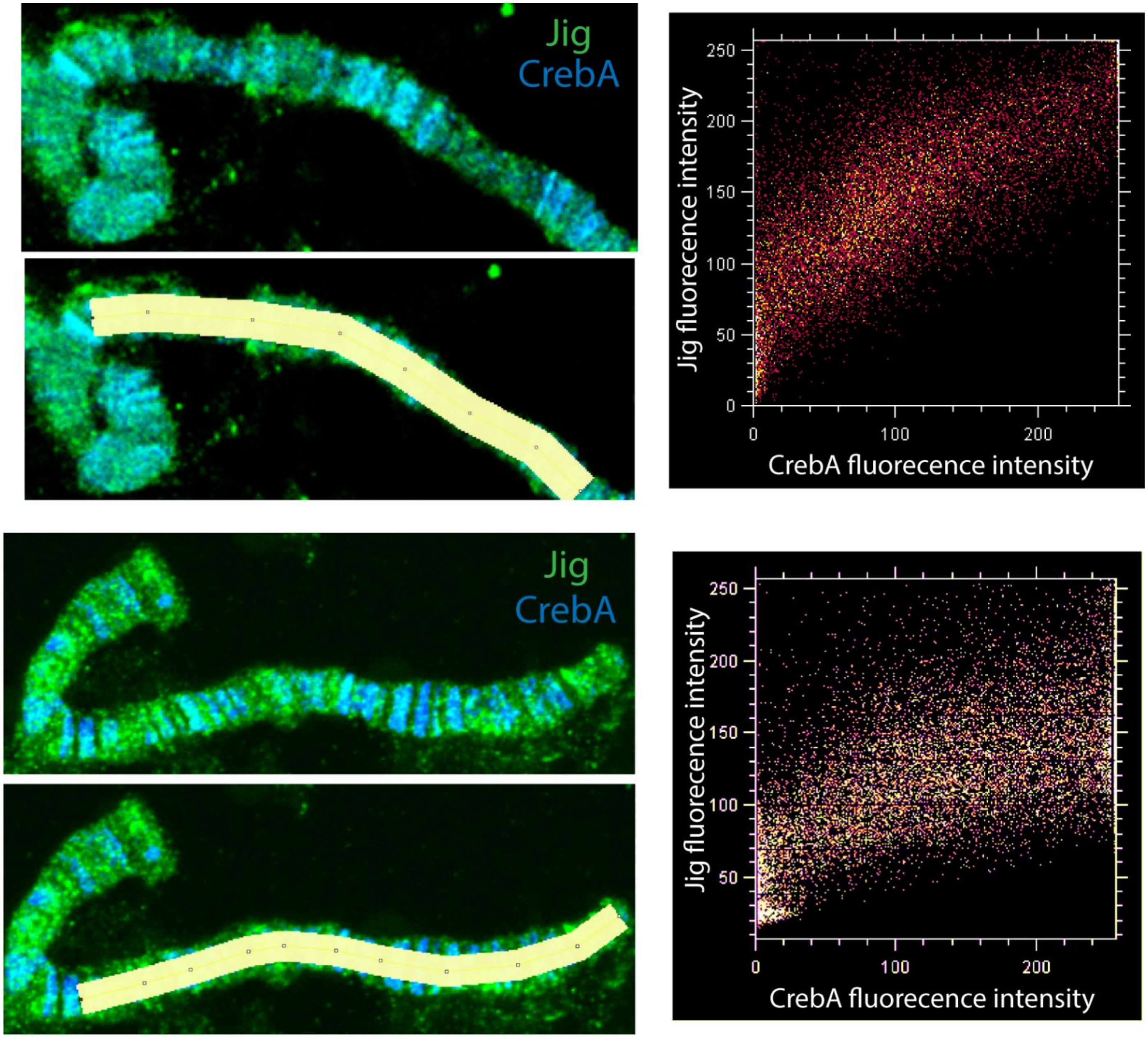

Supplemental Figure S6. Jig colocalizes with CrebA on chromatin. Left images show how region of interest (ROI) were set up to investigate the colocalization between CrebA and Jig. Right images represent a scatterplot of the fluorescence intensity of CrebA (X-axis) and Jig (Y-axis). The Pearson correlation coefficients are respectively 0.7908 (Top) and 0.8103 (bottom), suggesting that Jig and CrebA colocalize. This analysis was performed on multiple chromatin fragments that exhibit similar results.

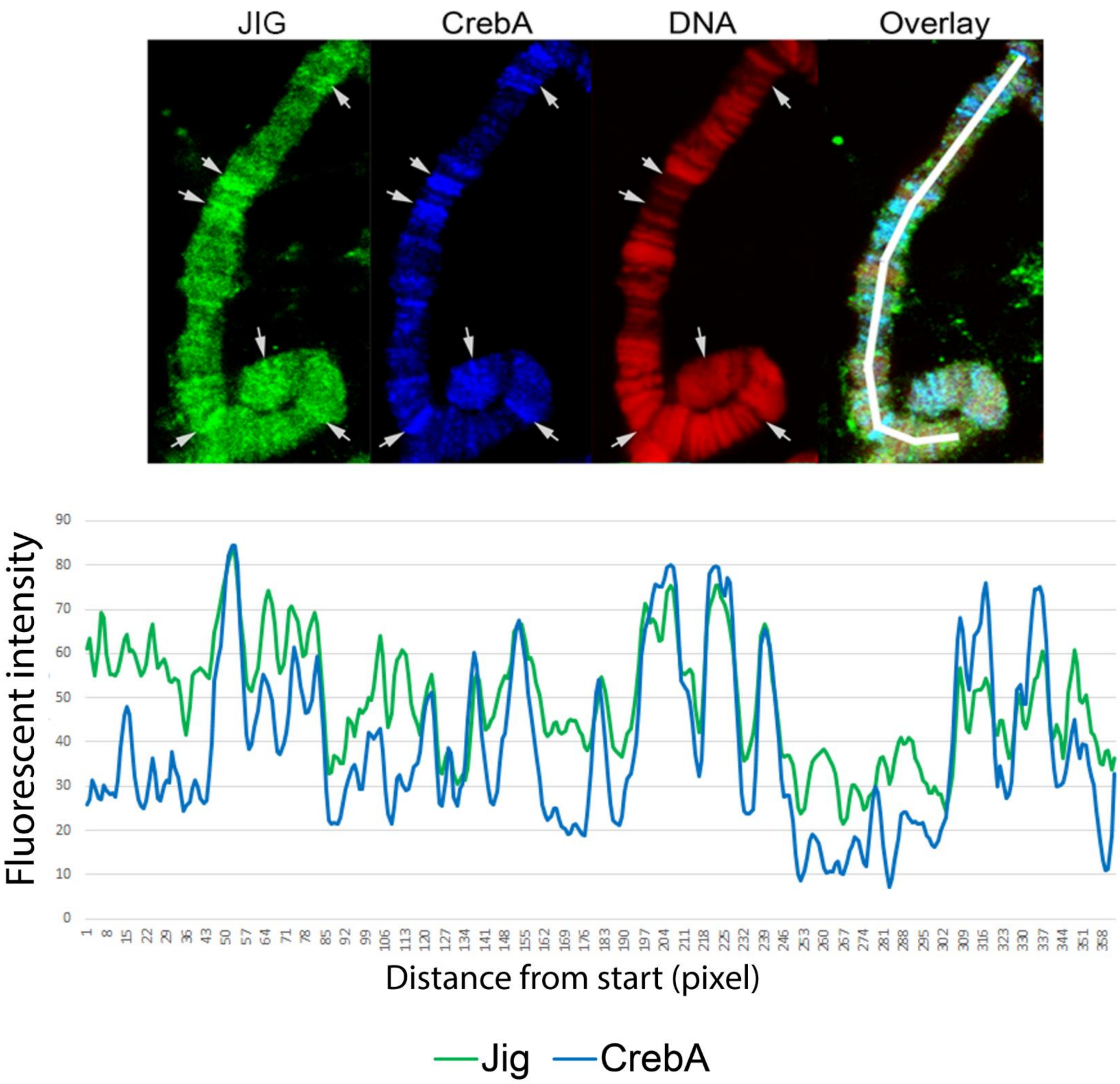

Supplemental Figure S7. The JIG and CREBA proteins fluorescence intensity along the polytene chromosome axis (white line in the overlay image) using with NIH image J software.

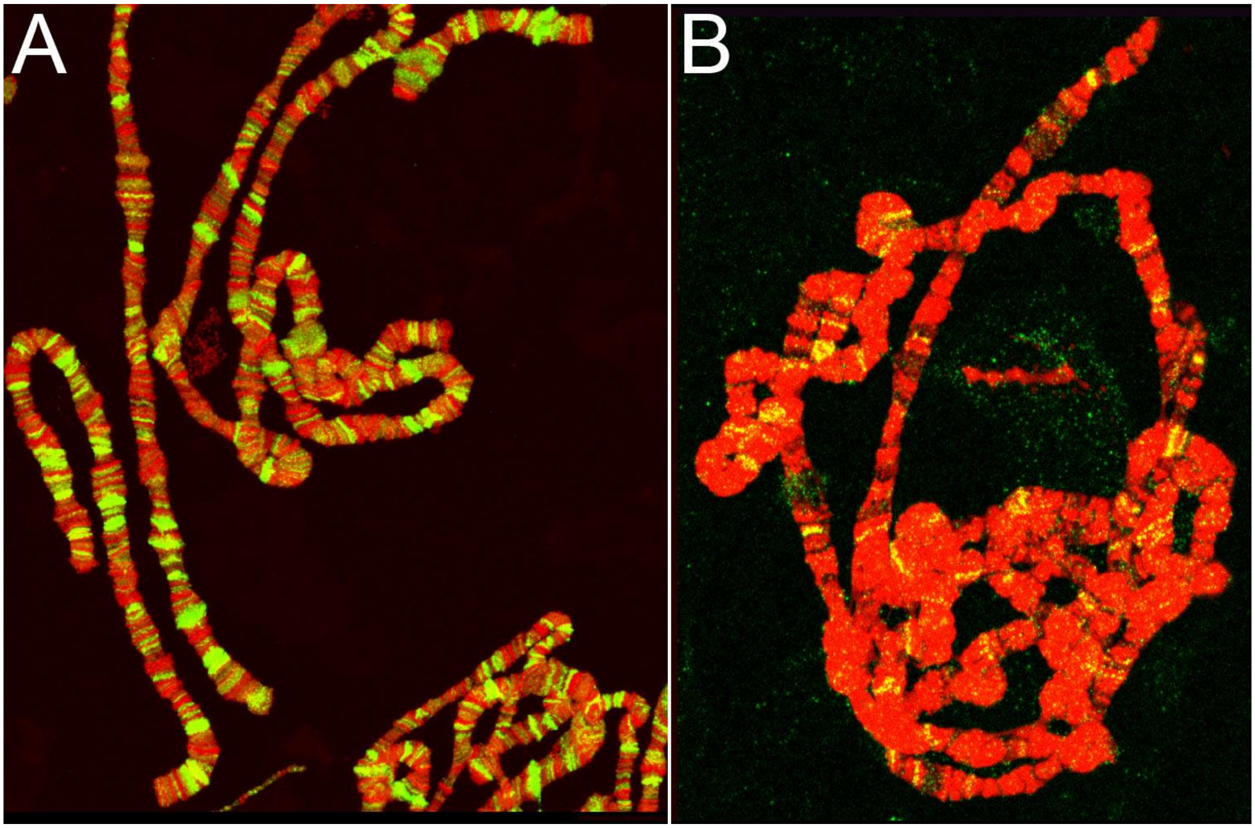

Supplemental Figure S8. JIG knockdown disrupts CREBA protein localization in chromatin. Salivary glands were dissected from wild type and JIG KD third instar Drosophila larvae, squashed and stained with anti-CREBA (green) antibody; DNA was detected using TOTO3 staining (red). CREBA protein binds ~ 300 loci in wild type Drosophila polytene chromosomes, while in JIG KD chromatin biding is dramatically diminished and only about 20 loci occupied.

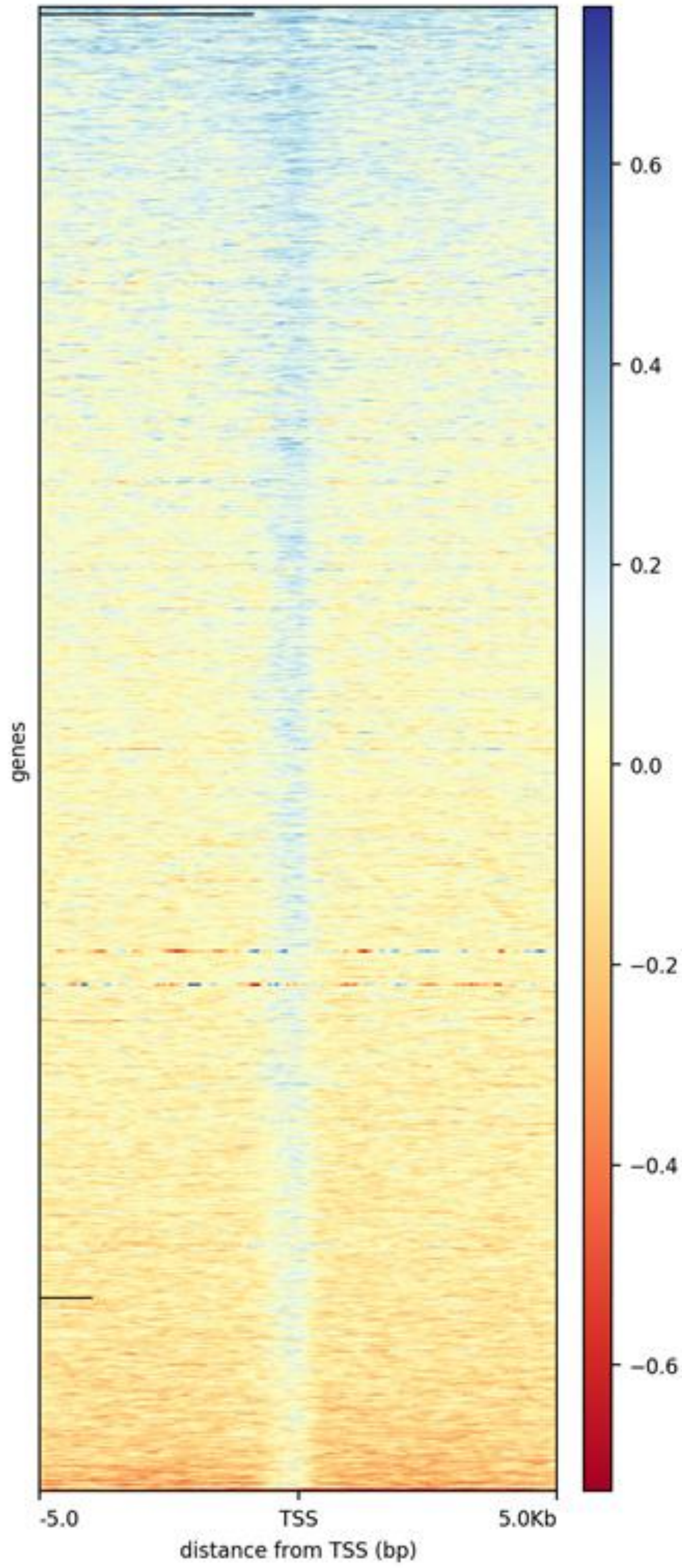

Supplementary Figure S9. Heatmap representing the distribution of JIG binding sites from -5 kb to +5kb in relation to TSS. The Y-axis represents the ratio of genes in JIG sample compared to control in logarithmic scale. JIG mostly binds to the promoter region.

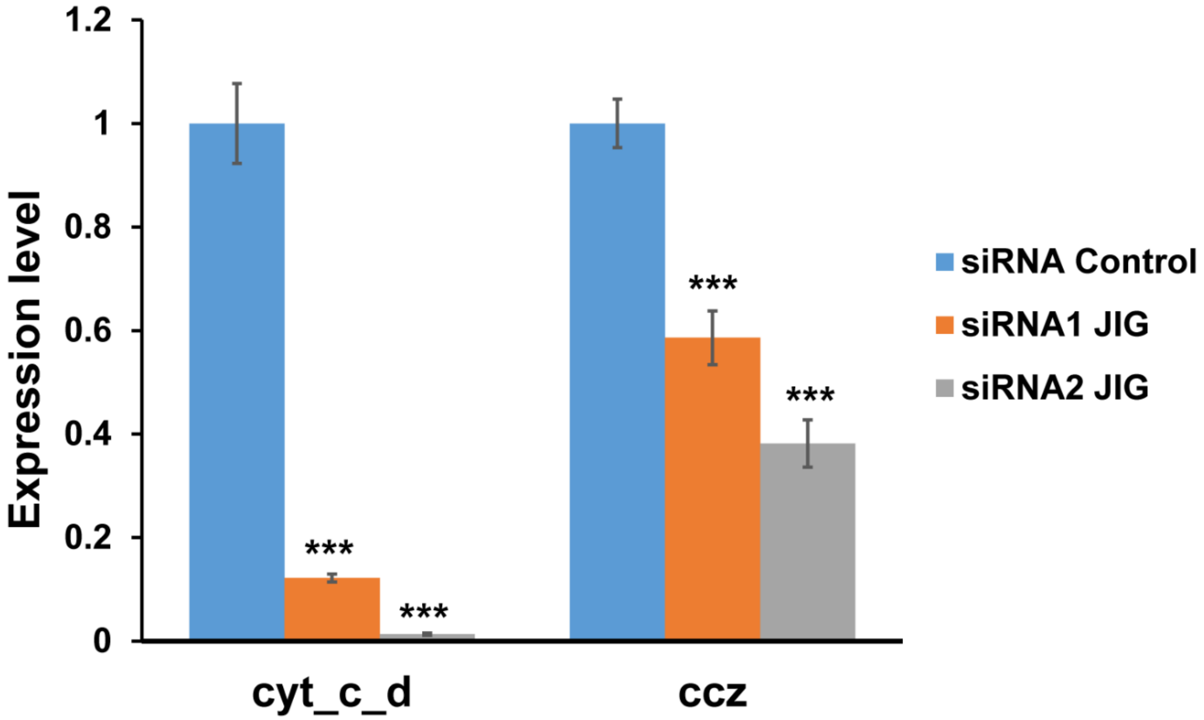

Supplemental Figure S10. Jig regulates the transcription of nuclear encoded mitochondrial protein. The expression level of Jig targets in Jig knockdown *Drosophila* larvae (orange and gray) compared to control (blue). Control expresses siRNA against LexA. Nuclear encoded mitochondrial proteins cyt_c_d and ccz are significantly downregulated when Jig is knocked down. This experiment was done in triplicates. *** = p-value < 0.01.
